# On the relationship and distinction between atomic density and coordination number in describing crystal defects

**DOI:** 10.1039/d6ra05403j

**Published:** 2026-10-23

**Authors:** Reza Darvishi Kamachali, Theophilus Wallis

**Affiliations:** a Federal Institute for Materials Research and Testing (BAM) 12205 Berlin Germany reza.kamachali@bam.de; b Institute of Materials Physics, University of Münster 48149 Münster Germany

## Abstract

Crystal defects are often described through broken atomic bonds, through counting the nearest neighbor coordination number. In this work, we highlight that the intrinsic heterogeneities in interatomic spacing due to a crystal defect, which is overlooked in broken-bond models, can decisively shape defect properties. We analyze excess free volume, energy, and entropy for a large set of BCC-Fe grain boundaries relaxed by molecular statics, and demonstrate that an atomic-density field, as a systematically coarse-grained field variable, shows much better correlations with the grain boundary properties. The atomic density field is shown to simultaneously capture bond depletion and spacing variations, thereby covering and unifying both structural and volumetric information. Our results establish the coarse-grained atomic density field as a principled surrogate for describing grain boundary thermodynamics, motivating the augmentation of broken-bond rules in predictive theories of interfacial energetics, excess properties, segregation, and phase behavior, and providing a direct bridge from atomistic to mesoscale models, with potential applications extending to other microstructural defects.

## Abstract 2

We establish the atomic density field as a principled surrogate for describing crystal defects.

## Graphical abstract

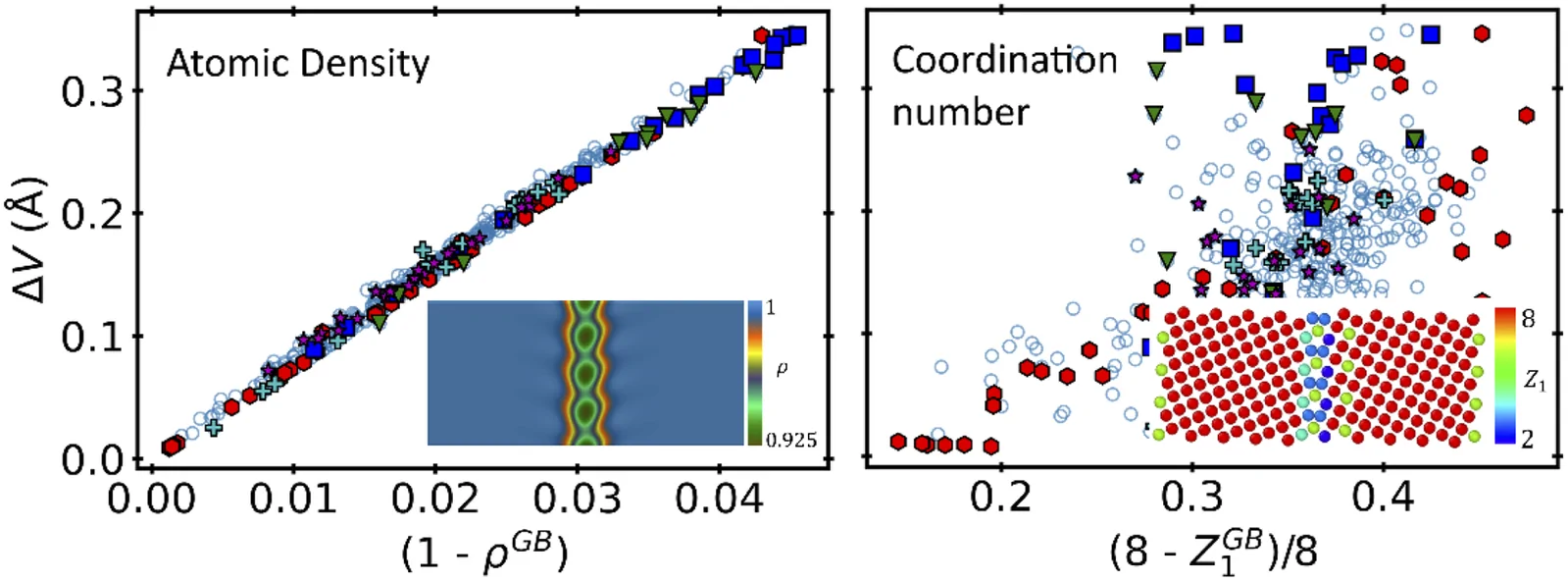

## 1 Introduction

One of the enduring concepts in modeling defects is the notion of broken bonds. This model was originally articulated in the context of calculating interface energy.^1,2^ This intuitive view treats interfaces as two-dimensional crystallographic defects in which the periodicity of the crystal lattice is disrupted and atomic bonding environments are redistributed. Within this framework, the reduction in atomic coordination at the boundary is used as a proxy for the energetic cost associated with grain boundary formation, and therefore, the coordination number—defined as the number of nearest neighbor atoms—plays a central role.

The coordination number serves as a structural descriptor in various atomistic and empirical models, such as the broken-bond rule for grain boundary energy,^3,4^ as well as in geometric and topological analyses of grain boundary networks.^5^ It has also been widely applied in models of solute segregation, where it forms the basis for understanding the local chemical potential shifts near interfaces. For instance, in bond-breaking or bond-counting models of segregation,^6,7^ solute atoms preferentially occupy under-coordinated sites at the grain boundary to reduce the system's energy. This principle underlies the McLean-type segregation isotherms^8^ and continues to inform modern statistical-mechanical and thermodynamic models of grain boundary segregation.^9,10^

The coordination number also has a longer conceptual route in coordination chemistry, where it denotes the number of ligands directly bound to a central atom and provides a basis for classifying local bonding geometries.^11,12^ More broadly, it remains actively used across bond analysis, atomistic modelling, high-throughput structural characterization, and materials informatics as a compact descriptor of local atomic environments.^13,14^ In particular, automated coordination-environment analyses now enable the robust assignment of coordination numbers and local geometries in large crystal-structure databases, including distorted inorganic environments.^12,14^ Closely related ideas also appear in modern local-environment descriptors and machine-learning structural representations, where the neighborhood of each atom is encoded for structure comparison, property prediction, and interatomic-potential construction.^15–18^

Despite its broad utility, the coordination-number approach has inherent limitations: it classifies atomic environments through discrete neighbor counts, but does not capture variations in interatomic spacing, local packing, or volumetric distortion. This limitation motivates the exploration of coarse-grained field descriptors, such as atomic density, that can generalize coordination-based models by retaining information on both neighbor multiplicity and spatial arrangement. In this work, we examine the relationship and distinction between coordination number and coarse-grained atomic density in describing crystal defects. Using 408 relaxed BCC-Fe grain boundaries, we show that atomic density retains information on both neighbor multiplicity and interatomic spacing, thereby capturing excess free volume, grain boundary energy, and configurational disorder more faithfully than coordination number alone.

## 2 Theoretical framework

In a series of recent studies, it has been proposed^19^ and extensively demonstrated^20–26^ that atomic density, formulated as a coarse-grained field variable, serves as a unifying variable, enabling the seamless integration of atomistic simulations, CALPHAD thermodynamics, and phase-field models for studying grain boundaries. The atomic density can be defined as1
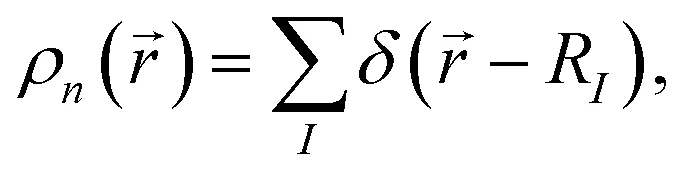
which sums over all atoms in a given system, with spatial coordinate *r⃑* and the position vectors of the atoms *R*_*I*_. The subscript *n* in *ρ*_*n*_ denotes the number density. The delta function can then be replaced by a three-dimensional Gaussian distribution function, thus giving2
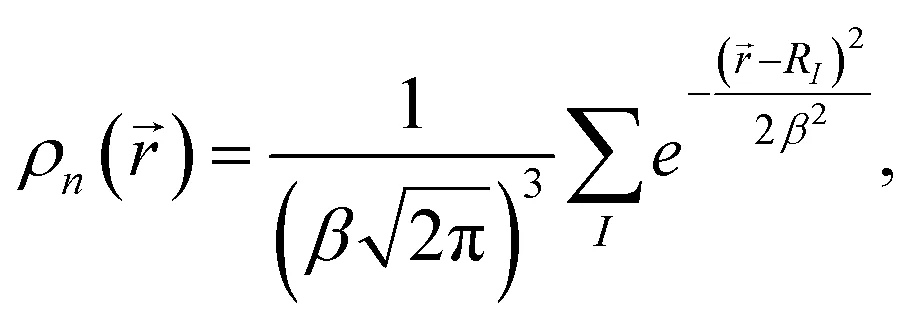
where *β* is the smearing radius. Considering a reference defect-free bulk system, we obtain the corresponding bulk atomic density3
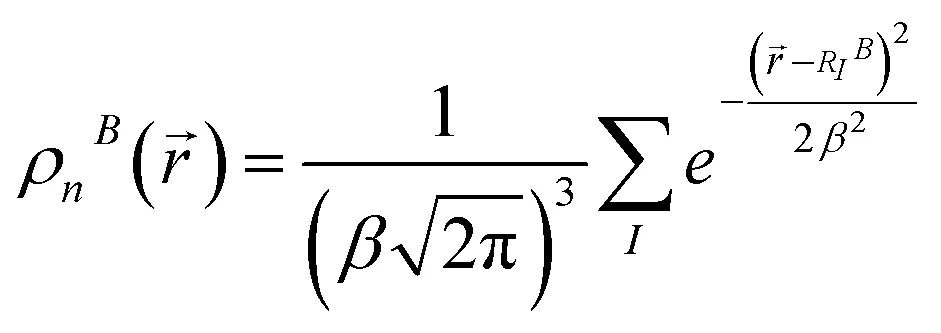
Here the superscript *B* refers to the defect-free bulk system. Given that an appropriate *β* value is chosen, *ρ*_*n*_^*B*^(*r⃑*) = *ρ*_*n*_^*B*^ is a constant value within the bulk domain. Thus, the final dimensionless atomic density field is defined as4
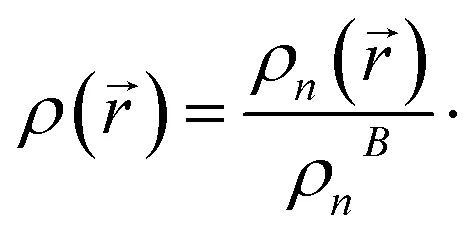


Although eqn (1)–(4) do not explicitly mention any relation with the coordination number, the sum in these equations which runs over all atoms in a given system inherently encodes information about the number of neighboring atoms. To make this point clear, let us denote the number of atomic neighbors in the *k*-th coordination shell by *Z*_*k*_, where *k* = 1, 2, 3, … corresponds to the first-, second-, third-nearest neighbors, and so on. The number of first-nearest neighbors, denoted as *Z*_1_, is then commonly referred to as the coordination number (a term originally coined from coordination chemistry).

In the reference bulk crystal, the neighboring shells are defined based on the peaks in the radial distribution function, with each shell corresponding to a characteristic distance from a reference atom. Using eqn (3) and the definitions outlined above, one can write the atomic density field in a defect-free bulk substance as5
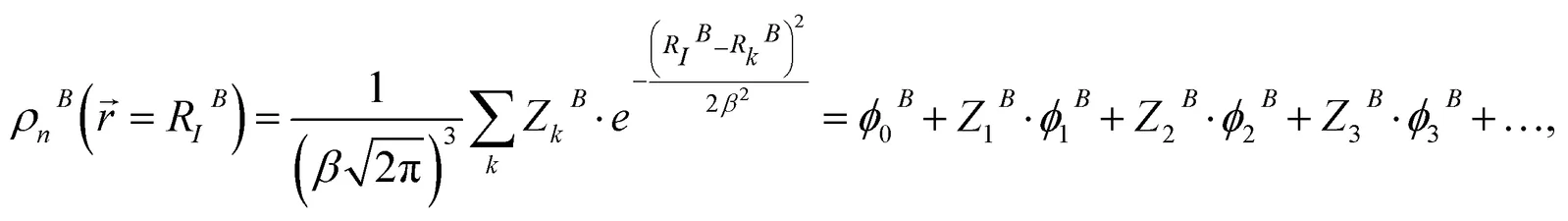
where the summation is restructured from individual atoms *I* to atoms in distinct coordination shells *k*, with *Z*_*k*_^*B*^ denoting the number of atoms in the *k*-th shell and *R*_*k*_^*B*^ denoting their equilibrium radial position (where |*R*_*I*_^*B*^ − *R*_*k*_^*B*^| is the shell radius). For instance, in a perfect body-centered cubic (BCC) structure, the first (coordination) shell contains *Z*_1_^*B*^ = 8 atoms, while the second shell contains *Z*_2_^*B*^ = 6, and the third shell *Z*_3_^*B*^ = 12, located at increasing radial distances *R*_3_^*B*^ > *R*_2_^*B*^ > *R*_1_^*B*^. In eqn (5),6
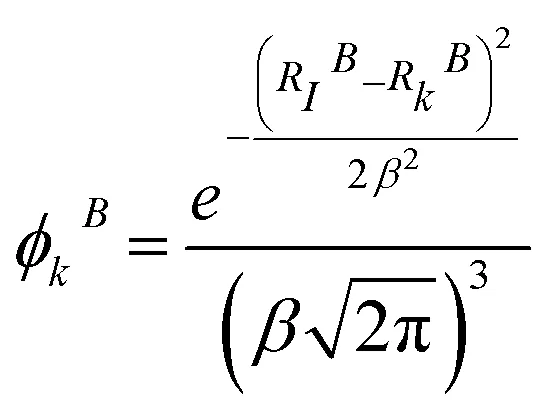
is the normalized Gaussian contribution from the *k*-th shell with respect to a reference atom at *R*_*I*_^*B*^. Here 
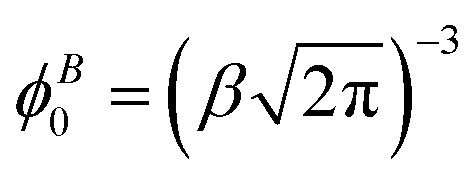
 corresponds to the center atom itself with *R*_*I*_^*B*^ = *R*_*k*_^*B*^. The physical unit of *ϕ*_*k*_s is [length]^−3^. The above formulation implicitly assumes crystal symmetries and isotropic atomic environments, as appropriate for a perfect crystal. Note that, for the sake of simplicity, eqn (5) is written only at atomic positions *r⃑* = *R*_*I*_^*B*^, yet the resulting value of the density field remains the same and a constant due to the translational symmetry of a defect-free crystal.

Referring now to disordered regions such as grain boundaries, the ideal shell structures become perturbed, resulting in local deviations in the number of neighboring atoms. This can be symbolically expressed as

7 *Z*_*k*_:*Z*_*k*_^*B*^ → {*Z*_*k*_}^GB^

where the brackets {… } indicate having a distribution and superscript GB denotes the grain boundary. This naturally induces variations in the coarse-grained atomic density field. Eqn (5) demonstrates that *ρ*(*r⃑*) directly reflects on the number of neighboring atoms.

Considering the leading first-shell term, one initially sees that the atomic density is proportional to the coordination number: *ρ*_*n*_^*B*^(*R*_*I*_) ≈ *Z*_1_^*B*^·*ϕ*_1_^*B*^. However, it is also readily clear that the interatomic spacing between neighboring atoms must also adjust accordingly to accommodate local structural (and in an alloy subsequently, chemical) inhomogeneities. In other words, not only does the discrete count of atoms in each coordination shell vary (eqn (7)) but so too do the radial distances and the corresponding kernel weights. Symbolically:

8 *R*_*k*_:*R*_*k*_^*B*^ → {*R*_*k*_}^GB^,

9 *ϕ*_*k*_:*ϕ*_*k*_^*B*^ → {*ϕ*_*k*_}^GB^.

The above analyses suggest that the coordination number (*Z*_1_), or even an extended set of neighbor counts including higher-order neighbor counts (*Z*_1_, *Z*_2_, *Z*_3_, …), is insufficient to fully characterize defect structures. Instead, it is the combined information on both the number and spatial proximity of neighboring atoms that defines the structural nature of a defect. These aspects are simultaneously captured by the atomic density field *ρ*(*r⃑*), such that regions with reduced coordination and/or expanded interatomic spacing—typically observed near disordered interfaces or other defects—manifest as local depletion in *ρ*(*r⃑*). Conversely, more densely coordinated and/or compressed regions correspond to local maxima. In contrast, coordination number metrics are insensitive to variations in interatomic spacing which are inherently captured in the kernel contributions (*ϕ*_1_, *ϕ*_2_, …).

*ρ*(*r⃑*) encodes not only the coordination number but also the local volumetric distortion, providing a more comprehensive representation of the atomic environment than coordination numbers alone—even when considering multiple neighboring shells. This enriched descriptive power is crucial for accurately capturing grain boundary structure, excess free volume, and long-range elastic fields, and is therefore essential for correctly computing the associated energy and entropy of the defect.

In the following, we confront the two descriptors with data. We analyze a comprehensive set of 408 distinct BCC-Fe grain boundaries (tilt, twist and mixed), generated and relaxed by molecular statics.^27^ Recent analyses^28,29^ show that the grain boundary density, defined as the average atomic density at the grain boundary plane,

10 *ρ*^GB^ = 〈*ρ*(*r⃑*)〉_at the GB plane_,

serves as a representative scalar quantity for the overall change in the atomic density field associated with a grain boundary.

Analogous to *ρ*^GB^, we can define the coordination number for every atom and average it at the grain boundary plane

11 *Z*^GB^_1_ = 〈*Z*_1_(*R*_*I*_)〉_at the GB plane_,

giving a similar dimensionless value (8 − *Z*^GB^_1_)/8.

For each boundary, we evaluate the density deficit (1 − *ρ*^GB^) and the coordination deficit (8 − *Z*^GB^_1_)/8, see Methods. These are then compared against three independent grain boundary properties: the excess free volume per unit area Δ*V*, the grain boundary energy *γ*^GB^, and two measures of information configurational entropy per unit area—one obtained from the atomic-density field *S*_*ρ*_^GB^ and the other from shell descriptors, the sum entropy 
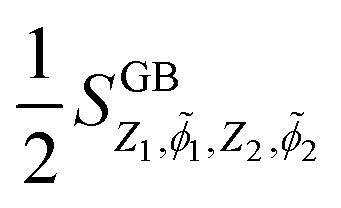
. Throughout, boundaries are grouped by parent-plane family for reference. We begin with representative examples (Fig. 1), then examine the joint variation of the two deficits (Fig. 2), followed by their relations to Δ*V* (Fig. 3) and *γ*^GB^ (Fig. 4). Finally, we discuss the entropic content (Fig. 5) and demonstrate the interesting equivalence between *S*^GB^_*ρ*_ and the sum entropy (Fig. 6).

**Fig. 1:**
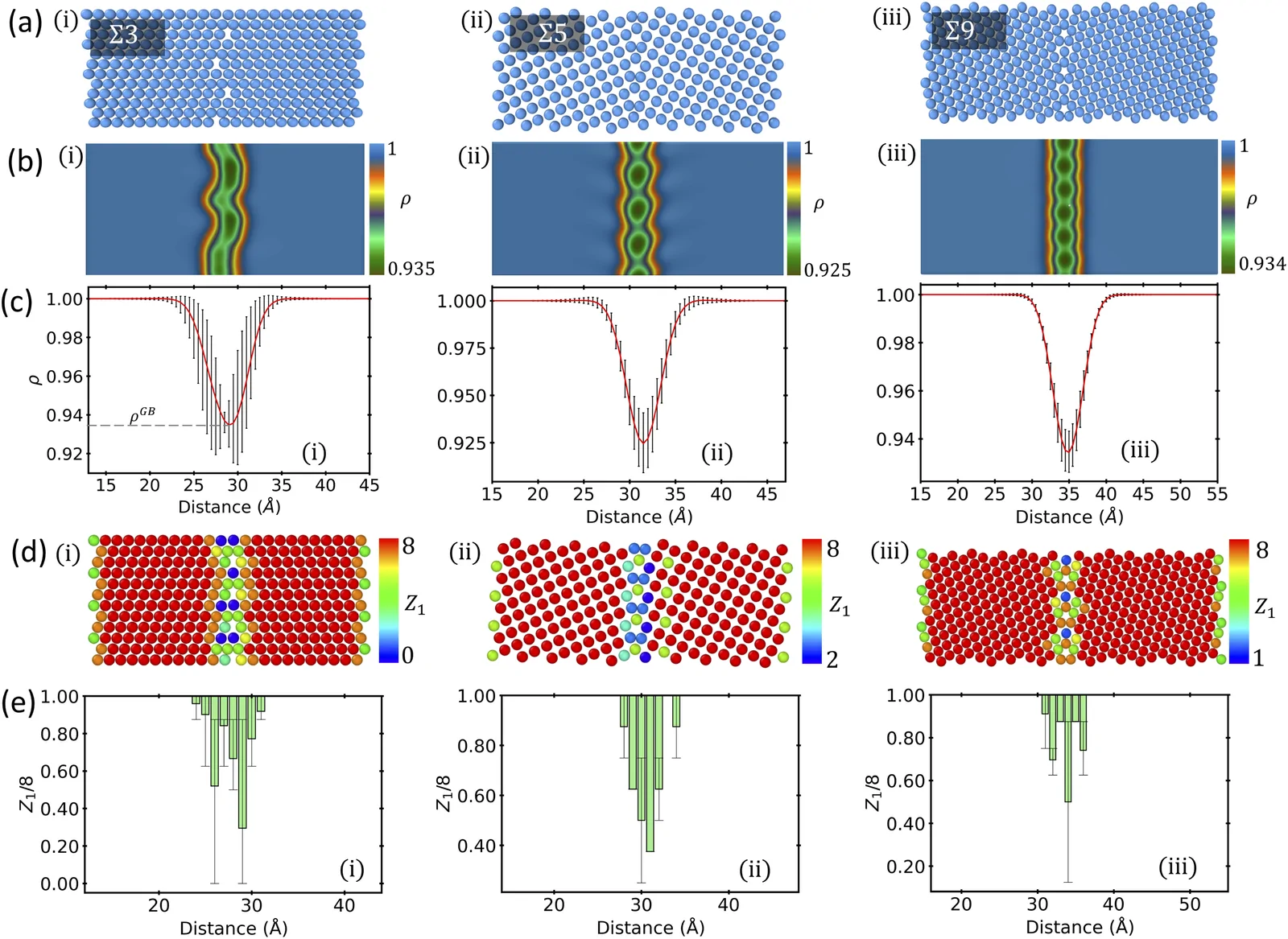
Representative grain boundary examples. (a) Atomistic structures for Σ3, Σ5, and Σ9 boundaries; (b) coarse-grained atomic density fields *ρ*(*r⃑*) showing depletion at the grain boundary plane; (c) density profiles *ρ*(*x*) across the grain boundary with *ρ*^GB^ marked; (d) atomic coloring by first coordination number *Z*_1_; (e) profiles of *Z*_1_(*x*)/8 across the grain boundary with reduced values at the interface. The error bars in (c) and (e) show the range of the depicted quantity in the corresponding *yz*-slice.

**Fig. 2:**
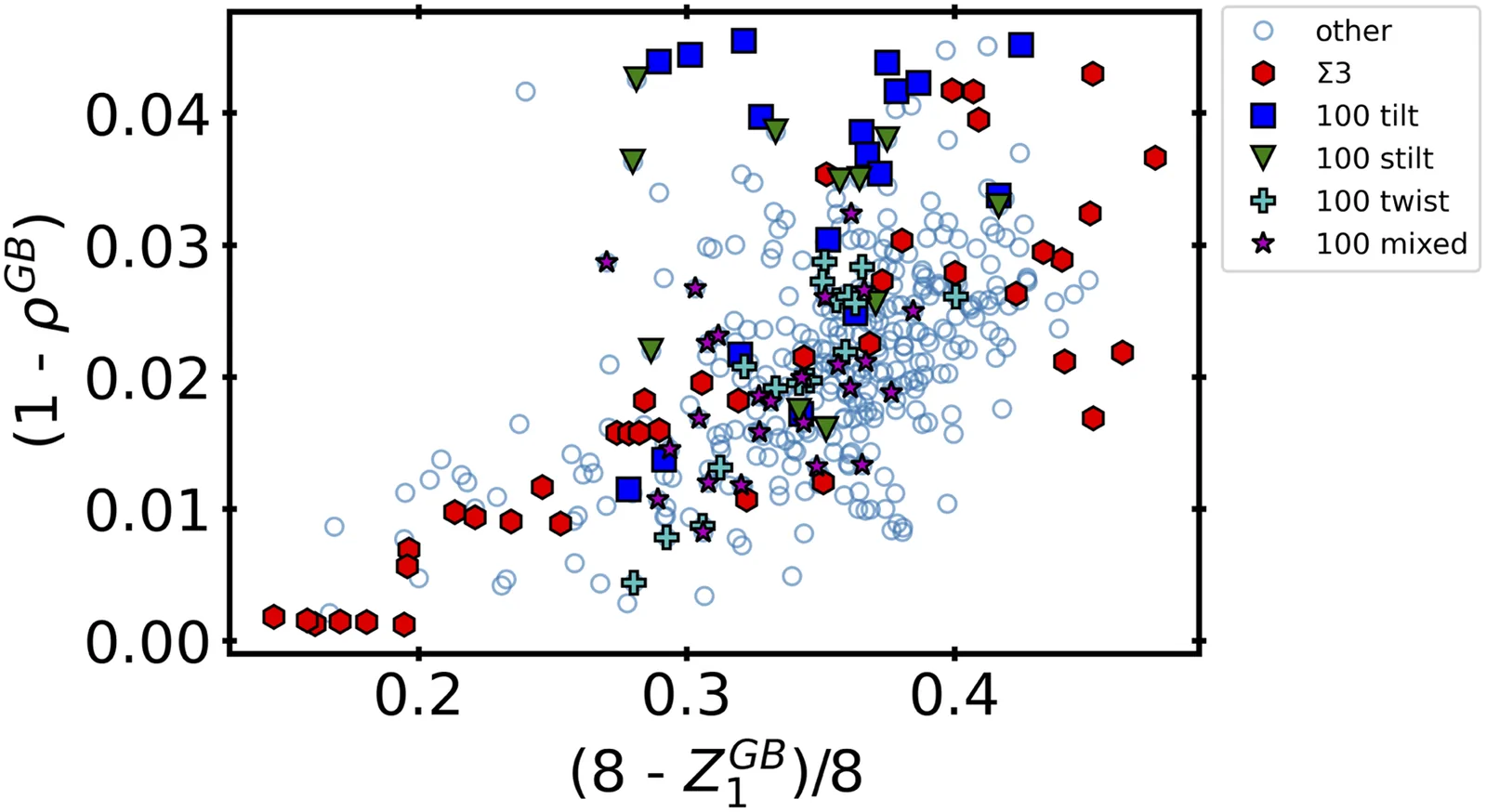
Correlation between density and coordination. Scatter plot of density deficit (1 − *ρ*^GB^) *versus* coordination deficit (8 − *Z*^GB^_1_)/8 at the grain boundary plane, across 408 BCC-Fe grain boundaries. Grain boundaries are classified by parent plane family, listed in the legend panel. This classification is the same in all following figures. The two descriptors exhibit a loose overall correlation, consistent with the contribution of coordination loss to atomic density deficit, but substantial scatter remains across the dataset. In particular, similar atomic density deficits can correspond to markedly different coordination deficits, demonstrating that *ρ*^GB^ and *Z*^GB^_1_ are related but non-equivalent structural descriptors.

**Fig. 3:**
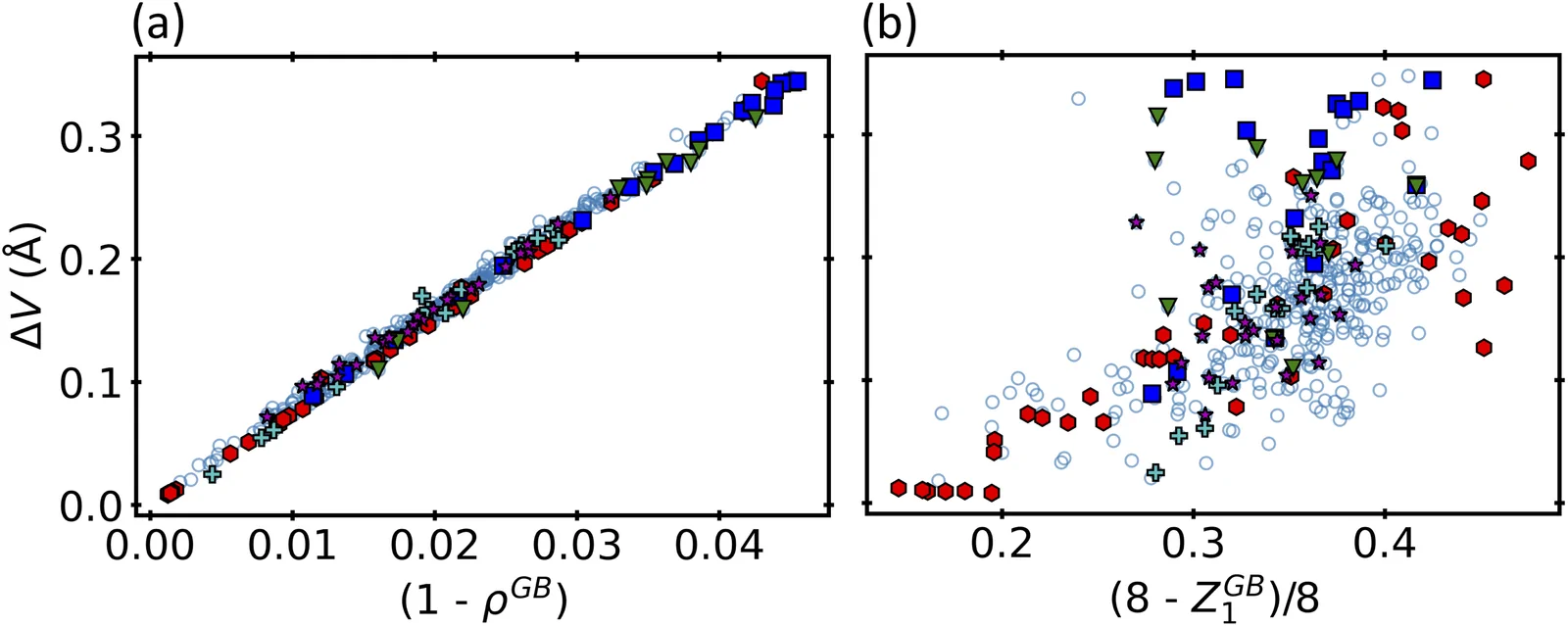
Grain boundary excess free volume *versus* density and coordination. (a) Excess free volume per unit area Δ*V*^GB^ collapses onto a near-perfect line with the density deficit (1 − *ρ*^GB^) across 408 BCC-Fe grain boundaries; (b) Δ*V*^GB^ shows a scattered relation with the coordination deficit (8 − *Z*^GB^_1_)/8, even for a given atomic plane family of grain boundaries.

**Fig. 4:**
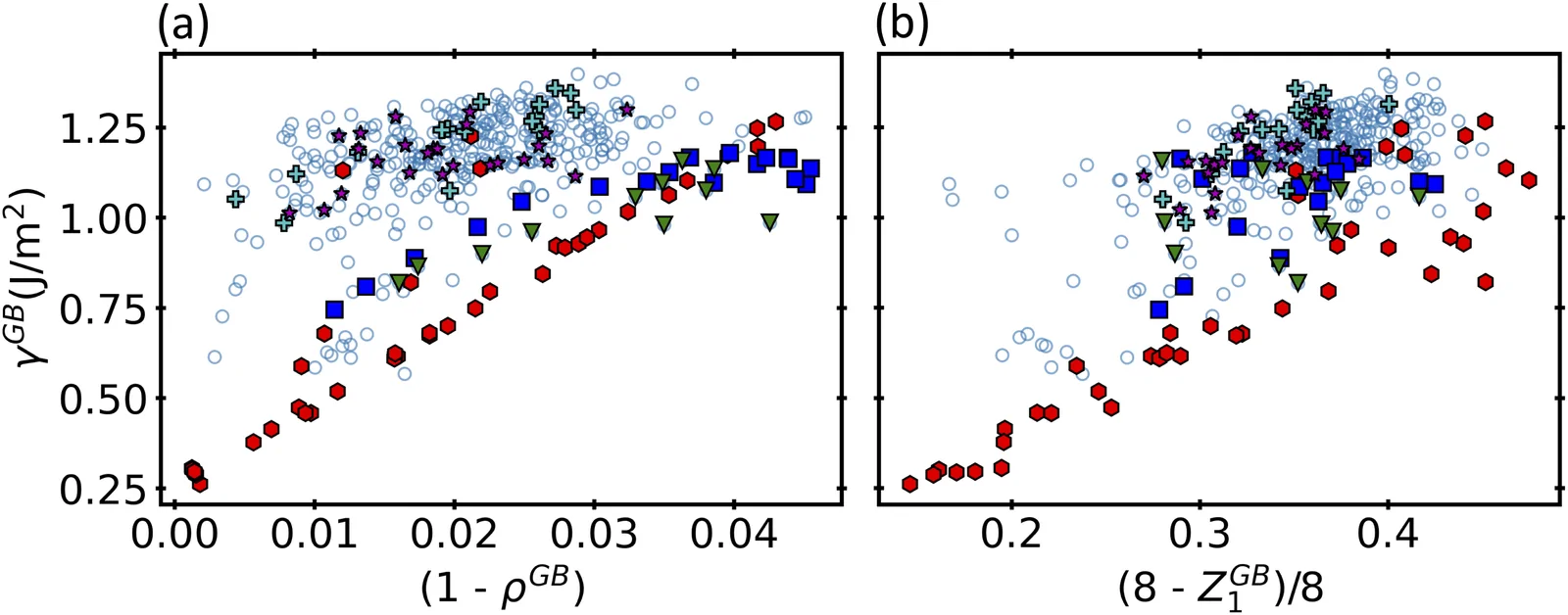
Grain boundary energy *versus* density and coordination. Grain boundary energy *γ*^GB^ exhibits a trend with both (a) density deficit (1 − *ρ*^GB^), and (b) coordination deficit (8 − *Z*^GB^_1_)/8. The different atomic plane families exhibit more distinct trends with the density deficit, whereas they show more scattered relations with the coordination deficit. Grain boundaries are classified by parent plane family, as indicated in the legend.

**Fig. 5:**
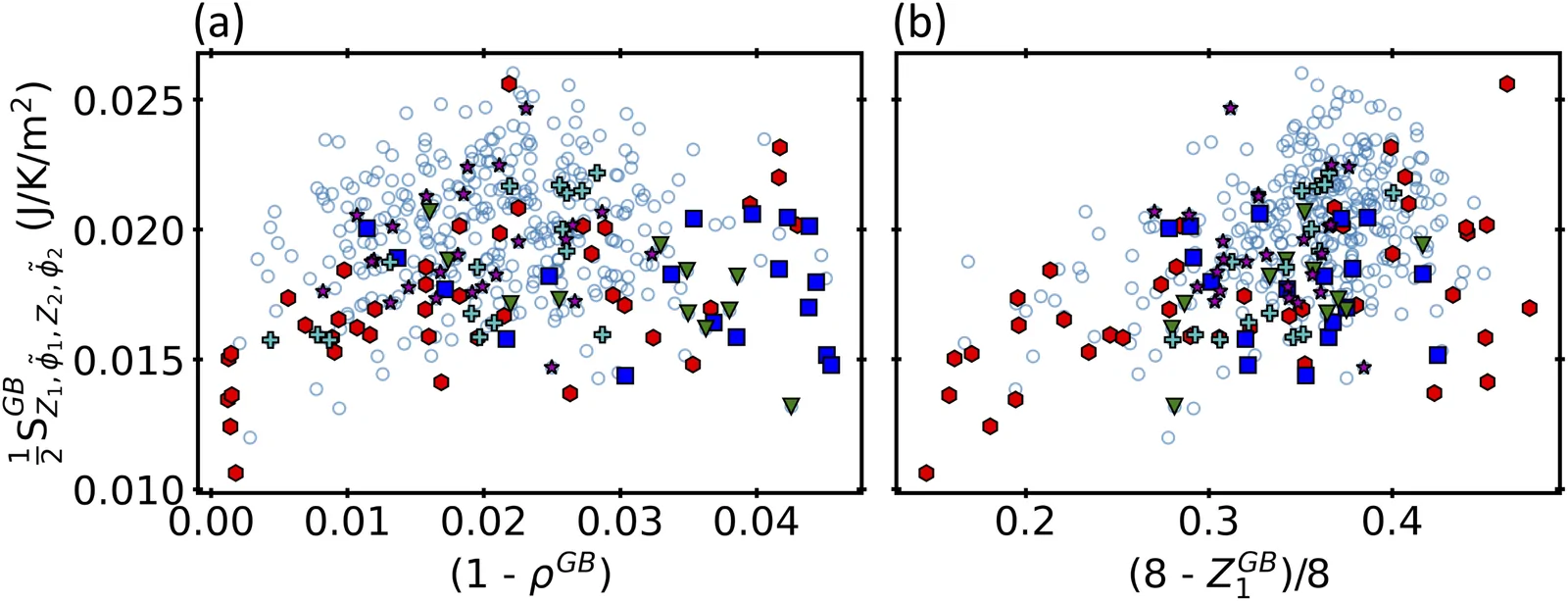
Configurational entropy *versus* density and coordination. (a) The sum configurational entropy per unit area 
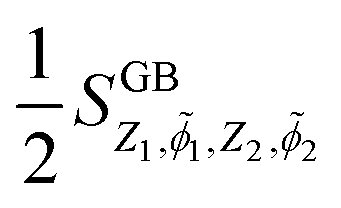
 exhibits an overall increasing trend with the density deficit (1 − *ρ*^GB^), reflecting growth of combined topological distance-related disorder with density depletion; (b) the same quantity exhibits an overall increasing trend with the coordination deficit (8 − *Z*^GB^_1_)/8. Considerable scatter is observed in both representations.

**Fig. 6:**
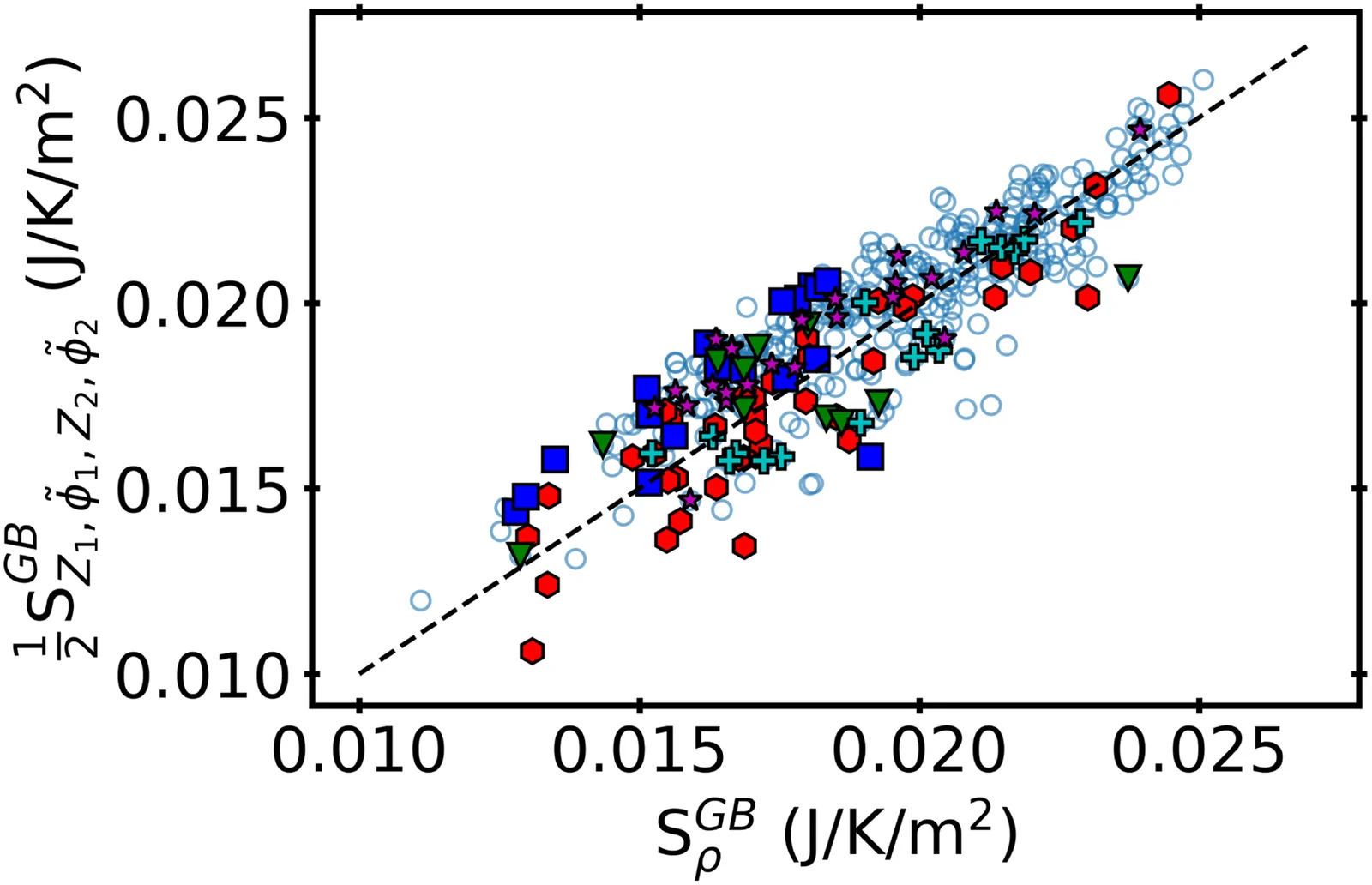
Equivalence of density and sum entropy. Areal configurational entropy from the density field *S*^GB^_*ρ*_, plotted against the sum entropy 
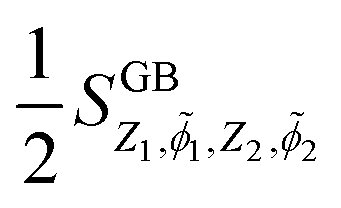
; data cluster tightly about the *y* = *x* line (dashed), indicating a near-unity slope and small intercept across 408 BCC-Fe grain boundaries, thereby validating the sum relation and underscoring the information captured by the atomic-density field beyond coordination counts.

## 3 Results and discussion

Fig. 1 shows representative grain boundary structures alongside the coarse-grained density fields and coordination maps, indicating that the planar-averaged density *ρ*(*x*) exhibits a clear depletion at the interface that robustly locates the GB, while the *Z*_1_ maps register coordination loss only where it occurs. All three Σ3, Σ5 and Σ9 boundaries show coincident reductions in both *ρ* and *Z*_1_, however, it is clear that the density field results in a consistent definition of the grain boundary region for all three examples, whereas the variations in coordination number are rather case-specific. More precisely, although both descriptors indicate the approximate locations of the interfaces shown in Fig. 1, they characterize the grain boundary region differently. For each of the Σ3, Σ5, and Σ9 boundaries, the averaged atomic density field defines a spatially continuous density depleted region relative to the bulk crystal, thereby providing a common criterion for delineating the interface. In contrast, the coordination number identifies only those atomic environments in which nearest neighbor connectivity changes. Individual atoms may retain the bulk BCC value *Z*_1_ = 8 while experiencing changes in interatomic spacing (see SI Fig. S2). Although coordination loss is present in the grain boundary averaged values across the investigated boundaries, coordination number alone does not capture the full range of structural variations reflected in the density field. Corresponding distribution profiles for the second-order neighbor count *Z*_2_ and the associated volumetric descriptors are shown in SI Fig. S1.

Fig. 2 shows the scatter of the density deficit (1 − *ρ*^GB^) against the coordination deficit (8 − *Z*^GB^_1_)/8 at the grain boundary plane for all grain boundaries. In all figures, grain boundaries are classified and colored based on their parent atomic planes. Fig. 2 primarily serves as an introductory comparison, demonstrating that the two structural descriptors are loosely related and non-equivalent across the grain boundary dataset. The loose overall trend is consistent with the fact that coordination loss contributes to atomic density deficit. Nevertheless, considerable scatter remains, *i.e.*, for a given density deficit (1 − *ρ*^GB^), the corresponding coordination deficit (8 − *Z*^GB^_1_)/8 spans a substantial range. Thus, coordination changes alone do not uniquely determine the atomic density response, consistent with the additional sensitivity of the atomic density field to variations in interatomic spacing. This distinction is examined more directly in Fig. 3 and 4 by comparing both descriptors with independently determined grain boundary excess properties.

Recently, we have demonstrated that grain boundary atomic density reveals a remarkable correlation with the excess free volume.^28^ This is a nontrivial result as the grain boundary density is the single (minimum) average density value at the grain boundary plane whereas the excess free volume is an integral property of the grain boundary. Fig. 3 contrasts the excess free volume per unit area, Δ*V*, with the density deficit (1 − *ρ*^GB^) and the coordination deficit (8 − *Z*^GB^_1_)/8. Panel (a) shows that Δ*V* collapses onto an almost perfectly linear trend with (1 − *ρ*^GB^) across all 408 boundaries, with only minor family-dependent spread, *i.e.* the single grain boundary plane quantity *ρ*^GB^ reliably predicts the integral excess volume.

By contrast, panel (b) exhibits a much broader relationship between Δ*V* and the coordination deficit. Although coordination deficit generally increases with excess volume, boundaries with comparable (8 − Z1GB)/8 can exhibit substantially different values of Δ*V*. This residual spread demonstrates that coordination loss alone does not determine the volumetric state of the interface. Variations in interatomic spacing and local dilation, which directly contribute to excess volume and are encoded by the density field, provide structural information beyond nearest neighbor counting. We note that Δ*V*, which is accessible through both atomistic calculations^28,30^ and experimental measurements^31^ is evaluated independently of both *ρ*^GB^ and the coordination number, thereby providing a common physical benchmark for comparing the two structural descriptors. Although the visual similarity between Fig. 2 and 3(b) is consistent with the strong correlation established in Fig. 3(a) and (b) is not derived from the other two plots. Rather, Fig. 3(a) and (b) directly compare how effectively the atomic density field and coordination number, respectively, describe the same independently determined grain boundary excess property.

Fig. 4 summarizes the energetic trends: *γ*^GB^ exhibits an overall trend with both the density deficit (1 − *ρ*^GB^) and the coordination deficit (8 − *Z*^GB^_1_)/8. However, the density-based representation reveals more distinct and clear family-dependent trends, whereas the coordination-based representation is considerably looser and highly dispersed. This suggests that, although both descriptors reflect structural changes relevant to the grain boundary energy, the density deficit retains additional information that better distinguishes the energetic response of different grain boundary families.

In order to quantify and discern the significance of the changes in interatomic spacing, we further our analyses by computing the entropy due to the disorder within a given system induced by a grain boundary. In principle, the relevant measure is the Shannon entropy, which arises from the combined topological (atomic coordination) and distance-related (variations in interatomic spacing) disorder within the grain boundary. These two aspects are respectively captured in eqn (5) where both coordination numbers and volumes are listed. Other degrees of disturbance can still be imagined, *e.g.*, the shape of closed volumes around every atom, which we omit from our entropy calculations. For a grain boundary region, truncating the atomic density formula to the first two shells, we can write

12 *ρ*_*n*_(*r⃑* = *R*_*I*_) ≈ *ϕ*_0_^*B*^ + *Z*_1_(*R*_*I*_)·*

<svg xmlns="http://www.w3.org/2000/svg" version="1.0" width="12.266667pt" height="16.000000pt" viewBox="0 0 12.266667 16.000000" preserveAspectRatio="xMidYMid meet"><metadata>
Created by potrace 1.16, written by Peter Selinger 2001-2019
</metadata><g transform="translate(1.000000,15.000000) scale(0.011667,-0.011667)" fill="currentColor" stroke="none"><path d="M320 1120 l0 -80 40 0 40 0 0 40 0 40 80 0 80 0 0 -40 0 -40 120 0 120 0 0 80 0 80 -40 0 -40 0 0 -40 0 -40 -80 0 -80 0 0 40 0 40 -120 0 -120 0 0 -80z M560 920 l0 -40 -40 0 -40 0 0 -80 0 -80 -120 0 -120 0 0 -40 0 -40 -40 0 -40 0 0 -80 0 -80 -40 0 -40 0 0 -120 0 -120 40 0 40 0 0 -40 0 -40 80 0 80 0 0 -40 0 -40 -40 0 -40 0 0 -40 0 -40 40 0 40 0 0 40 0 40 40 0 40 0 0 40 0 40 40 0 40 0 0 40 0 40 40 0 40 0 0 40 0 40 40 0 40 0 0 80 0 80 40 0 40 0 0 80 0 80 -40 0 -40 0 0 40 0 40 -40 0 -40 0 0 80 0 80 40 0 40 0 0 40 0 40 -40 0 -40 0 0 -40z m-160 -360 l0 -80 40 0 40 0 0 80 0 80 80 0 80 0 0 -80 0 -80 -40 0 -40 0 0 -80 0 -80 -40 0 -40 0 0 -40 0 -40 -40 0 -40 0 0 120 0 120 -40 0 -40 0 0 -120 0 -120 -80 0 -80 0 0 80 0 80 40 0 40 0 0 80 0 80 40 0 40 0 0 40 0 40 40 0 40 0 0 -80z"/></g></svg>


*_1_(*R*_*I*_) + *Z*_2_(*R*_*I*_)·**(*R*_*I*_)

where *ρ*_*n*_(*r⃑* = *R*_*I*_) is computed based on eqn (1)–(4) and the right-hand side can be obtained for each atom such that *φ̃*_1_ and *φ̃*_2_ are13
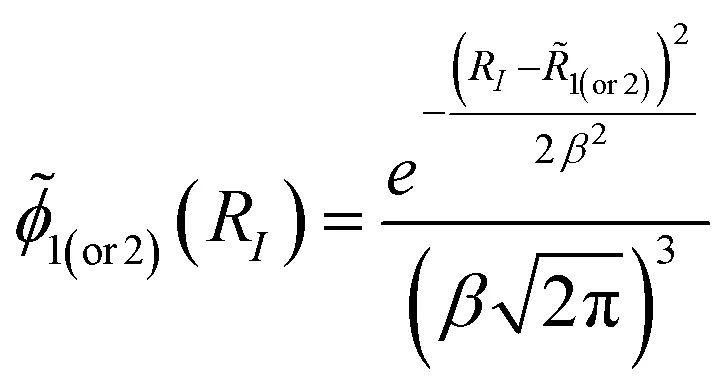
with *R̃*_1_ and *R̃*_2_ being effective radii computed based on the respective effective volume of the first and second coordination shell around a given atom. Note that *ϕ*_0_^B^ is a constant and plays no role in computing the entropic contributions.

For the purpose of our comparison, we compute the entropy in two ways: once from the spatial distribution of the atomic density field across the grain boundary *S*^GB^_*ρ*_, and once from per atom distributions of the descriptors *S*^GB^_*X*_, with *X*: *Z*_1_, *Z*_2_, *φ̃*_1_, *φ̃*_2_. See Methods for the precise definitions and procedure. From the descriptor entropies, the sum entropy reads14
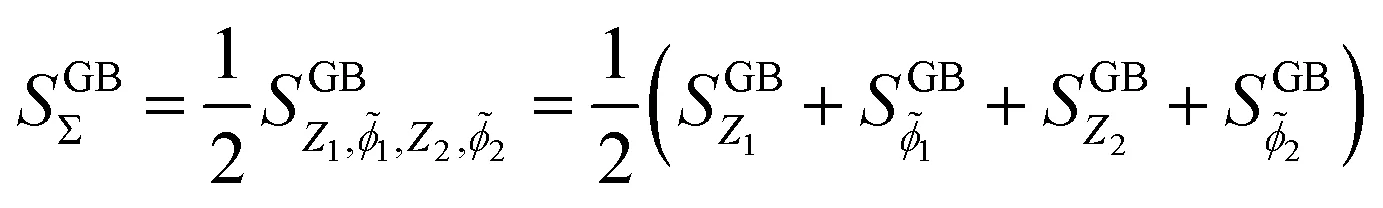


See Methods section for details on construction of the individual entropies and the derivation of sum entropy. The configurational entropy calculated here is an information-theoretic measure derived from the probability distribution of the density descriptor. It quantifies the configurational or spatial diversity of the sampled grain boundary environments and should not be identified with the complete thermodynamic entropy of the grain boundary. Accounting for the full thermodynamic entropy would require exploration of alternative grain boundary microstates.

Fig. 5 presents the sum entropy 
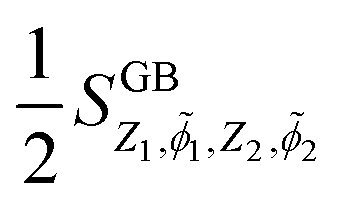
 per unit area against the two grain boundary plane descriptors. The results show an overall increasing trend of the sum entropy with both the density deficit (1 − *ρ*^GB^) and the coordination deficit (8 − *Z*^GB^_1_)/8, although considerable scatter is observed in both representations. This overall behavior is consistent with increasing structural disorder as the grain boundary structure deviates from the bulk environment. The contributions due to individual descriptors (
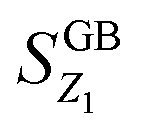
, 
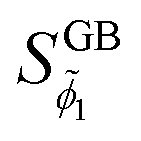
, 
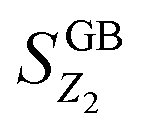
, 
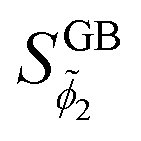
) are shown in Fig. S3 and S4 in the SI. Interestingly, we find that the entropy contributions due to coordination deficiency (
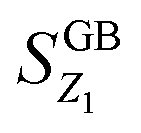
, 
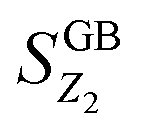
) are much smaller than the entropy due to disturbance in the atomic spacing (
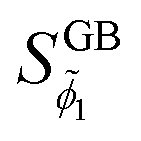
, 
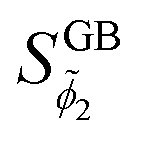
). The latter significantly dominates, by a factor of ∼5 to 10, varying slightly from grain boundary to grain boundary. This decoupling confirms that coordination counts alone underestimate the configurational disorder when spacing fluctuations dominate.

Fig. 6 compares the sum entropy from coordination descriptors *versus* the entropy directly computed from the atomic density field. We find that *S*^GB^_*ρ*_ aligns tightly with 
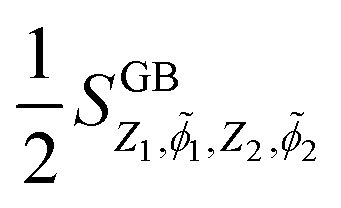
 along the *y* = *x* line, with a near-unity slope and a small intercept across all families. This is a principal result of our study, revealing the numerical equivalence of the density-based entropy and the sum entropy, thereby confirming that the atomic-density field compactly captures the joint topological distance-related disorder encoded by the shell descriptors. In comparison, the first neighbor coordination entropy 
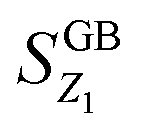
 exhibits an overall loose relationship with the sum entropy, but with appreciable scatter (SI Fig. S6). A comparison between the two measures of information configurational entropy per unit area and the grain boundary energy is shown in SI Fig. S5, showing a different presentation of this equivalency.

A central feature of the present results is that coordination loss and density depletion are loosely related, but not uniquely so. This is physically expected: changes in nearest neighbor multiplicity directly affect the local atomic density, while the density field additionally responds to changes in the spatial separation of those neighbors. Consequently, two grain boundaries with similar coordination deficits may exhibit different density deficits, excess volumes, and energies because their atomic environments accommodate structural distortion through different combinations of connectivity changes and interatomic displacements. The differences between the density- and coordination-based trends observed in Fig. 2–5 therefore reflect the distinction between a discrete topological descriptor and a field variable that additionally retains geometrical information.

We find that the density framework is agnostic to grain boundary character and thus captures features that are generic across classes. Considering low-angle boundaries, where the structure is well-described as an array of dislocations with long-range elastic fields, coordination changes may remain localized around dislocation cores, whereas the accompanying dilatational/compressional strain fields extend over a broader region. The density field is sensitive to both contributions. Conversely, in more disordered or high-angle boundaries, where substantial local volume changes and topology disruptions occur, both *Z*_1_ and *ρ* vary, but the density field integrates spacing heterogeneity and excess volume more faithfully, yielding the tight trends with Δ*V*, *γ*^GB^, and the growth of configurational entropy.

Taken together, the results show that *ρ*(*r⃑*) unifies long-range elastic effects (with minimal coordination change) and strongly disordered cores (with both topology and spacing changes), thereby providing a single state variable that is predictive across the full grain boundary spectrum. These aspects motivate that the atomic density field parameter can be very useful in studying the mechanical^32–34^ or electrical^35^ response of grain boundaries, in the same spirit of the grain boundary excess volume. For liquids, Hong and Liu proposed a one-trajectory molecular dynamics framework that evaluates configurational entropy directly from nearest neighbor statistics and vibrational entropy from the phonon density of states.^36^ They showed that vibrational entropy provides the dominant contribution even in liquids, while their coordination-based configurational entropy remains comparatively small. This supports our emphasis on spacing-sensitive (volumetric) descriptors of atomic disorder, as vibrational entropy reflects the curvature of the interatomic potential and is therefore highly sensitive to interatomic spacing. Another remarkable aspect of *ρ*(*r⃑*) and *ρ*^GB^ is their potential to link with the experimental measurements: quantitative routes to measure local atomic density at (deformation) interfaces are developed by combining HAADF-STEM with EELS thickness calibration and 4D-STEM with machine learning techniques.^37–40^ Independent radiotracer experiments revealed orders-of-magnitude enhancement of atomic diffusion inside free-volume-rich short-circuit paths^41^ which can also be linked with the atomic density field.

The distinction between coordination and interatomic spacing may also have implications beyond grain boundary thermodynamics. Coordination-based descriptors have been extensively employed to rationalize adsorption and catalytic activity at surfaces,^42–44^ while strain-induced changes in interatomic spacing are known to modify adsorption energetics and surface reactivity.^45^ The latter observation is particularly relevant to the present framework, as the coarse-grained density remains sensitive to changes in local atomic spacing even for local atomic environments in which the coordination number remains unchanged. More generally, the heterogeneous atomic environments associated with grain boundaries are known to influence other defect mediated phenomena, including solute segregation and diffusion.^46,47^ This suggests that a descriptor simultaneously sensitive to neighbor multiplicity and spatial rearrangement may provide a complementary structural description for relating local defect structure to adsorption, segregation, diffusion, and catalytic activity. Establishing such relationships quantitatively, however, requires property-specific investigations and remains an interesting direction for future work.

## 4 Conclusion and outlook

To summarize, the above analyses show that a coarse-grained atomic density field *ρ*(*r⃑*), captures the essential grain boundary physics and more completely than coordination number. While *Z*_1_ registers broken bonds, *ρ* embeds both topology (neighbor multiplicity) and spacing (local dilation/compression). Across the 408 BCC-Fe boundaries, the coordination deficit is loosely related to the atomic density deficit, confirming the expected contribution of coordination loss to the atomic density field. Importantly, however, the relationship remains substantially scattered, demonstrating that the two descriptors are not interchangeable. This distinction becomes particularly clear for the excess free volume: (1 − *ρ*^GB^) collapses Δ*V* onto an almost perfect linear trend, whereas (8 − *Z*^GB^_1_)/8 exhibits a much broader relationship. The results therefore reinforce the central conclusion that coordination loss captures only part of the structural information associated with a grain boundary, while the density field additionally retains the effects of interatomic spacing and local volumetric distortion.

A second conceptual outcome concerns configurational disorder. We show that the density-based entropy *S*^GB^_*ρ*_ is able to track the sum entropy 
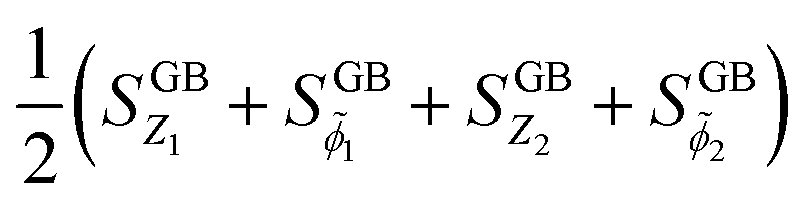
, nearly one-to-one within the approximations adopted here. This close numerical agreement suggests that *ρ* provides a compact, effectively lossless proxy for the joint topological distance-related disorder. In entropy (information) terms, the strong in-shell correlations (between *Z*_*k*_ and *φ̃*_*k*_) imply substantial redundancy; the observed one-half prefactor reflects an effective two-mode structure dominated by the first two shells. Thus, *ρ* appears to summarize what matters for grain boundary thermodynamics without proliferating descriptors.

Practically, our results argue for augmenting broken-bond rules with density-based descriptors in models of grain boundary energetics, excess properties, and segregation thermodynamics. Because the atomic density can be both a mean field parameter (*ρ*^GB^) or a full field (*ρ*(*r⃑*)), it interfaces naturally with continuum treatments (*e.g.*, CALPHAD and phase-field) and can, additionally and consistently, carry elastic fields and volumetric excess. The subsumption of coordination number, interatomic spacing, excess volume and isotropic elasticity within a single density-based framework suggests that *ρ*(*r⃑*) is a convenient surrogate and a candidate descriptor of crystal disorder. Limitations remain: our dataset is BCC-Fe at 0 K (molecular statics), the coarse-graining length *β* sets resolution and must be chosen judiciously, and the entropies computed are specific (minimum-energy) configurational measures rather than full thermodynamic entropies. Nonetheless, the robustness of the trends across orientations and families of grain boundaries suggests that the density framework is structurally generic.

From a chemistry perspective, the current discussion connects to the broader question of how local bonding environments are represented in atomistic, electronic-structure, and mesoscale descriptions of defective materials. Bond-order analyses, coordination-based descriptors, local charge-density measures, free-volume concepts, and machine-learning structural fingerprints all seek to quantify how local atomic environments depart from an ideal reference structure. Such departures are expected around all sorts of crystal defects. In all these cases, the relevant structural change is rarely limited to missing or weakened bonds; it also involves changes in bond lengths, local packing, volumetric distortion, elastic strain, and electronic bonding character. This suggests that chemically intuitive descriptors based on coordination should be complemented by the field-like descriptors that retain information on both neighbor multiplicity and spatial arrangement. In this sense, the atomic density field may provide a general bridge between local bonding analysis, defect thermodynamics, and mesoscale descriptions of structure evolution.

The quantitative conclusions presented here have been established for the investigated dataset of relaxed BCC-Fe grain boundaries. The underlying distinction between neighbor multiplicity and interatomic spacing follows from the definitions of the coordination number and coarse-grained density and may therefore extend to other crystal structures and defect types. However, the quantitative performance of the density descriptor, particularly its relationships with defect excess properties, remains to be established for FCC and HCP crystals and for other defects such as surfaces, dislocations, and interfaces. Extension to multi-component systems may additionally require species dependent weighting and/or coarse-graining length scales to account for chemically and structurally distinct atomic environments. Complementary experimental investigations, building on approaches such as those in ref. 35 and 37–41, could further examine atomic density deficits and excess volumes using diffraction, positron annihilation, high-resolution microscopy, and related atomic-scale characterization techniques. Such studies would provide important tests of the proposed framework and help bridge atomistic descriptions, mesoscale models, and experimental characterization.

## 5 Methods and computational details

### 5.1 Atomistic simulation dataset

We analyze a set of 408 distinct grain boundaries (GBs) in BCC-Fe generated and minimized following established protocols.^27,28^ Bicrystals are constructed with periodicity along the grain boundary(*y* and *z* axes) and non-periodicity with sufficient padding normal to the grain boundary(*x* axis) to suppress image interactions. After rigid-body scans and in-plane relaxations, structures are relaxed to a force and energy tolerance by conjugate-gradient minimization. Grain boundary energies and excess free volumes are computed using the atomistic simulations, for each grain boundary, and both are per unit area. All coarse-graining and further analyses reported below are performed on the minimized configurations. Further details of structure generation and parameterization, as well as on the methods for coarse-graining the atomic density field, are provided in ref. 27 and 28; for the present study, we only summarize the definitions needed in the body of the text. For visualization, we group grain boundaries by parent plane families and special boundaries (*e.g.*, Σ3) following.^28^ Symbols and colors in the figures map consistently across panels; all scalars are shown with appropriate units or are dimensionless (for the atomic density and coordination number deficit at the grain boundary plane).

### 5.2 Computing atomic density and coordination number

In each simulation box and at the (equilibrium) position of each atom (*R*_*I*_), we compute the atomic density value *ρ*(*r⃑* = *R*_*I*_), the coordination number, *Z*_1_(*R*_*I*_), and the 2nd coordination number, *Z*_2_(*R*_*I*_). Note that *ρ*(*r⃑*) can be evaluated at any position in the system, whereas coordination numbers are defined only for atoms. The atomic density is computed using the coarse-graining approach described in relation to eqn (1)–(4), and the coordination properties are computed by OVITO post-process Python scripting. The coarse-grained atomic density computation is, of course, sensitive to the coarse-graining length (smearing radius *β* in the Gaussian distribution). This sensitivity is thoroughly discussed in 28. We use *β* = 2.4*r*_Fe_, with *r*_Fe_ = 1.26 Å being the atomic radius of iron. The grain boundary plane is defined as the location of the minimum of the planar-averaged density along the interface normal. We compute15
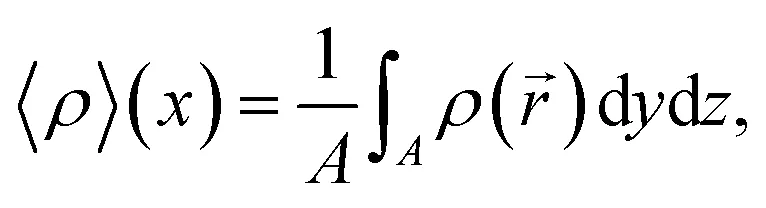
identify *x*_GB_ = arg·min_*x*_〈*ρ*〉(*x*), and evaluate grain boundary plane averages over a narrow slab of thickness *w* centered at *x*_GB_,16
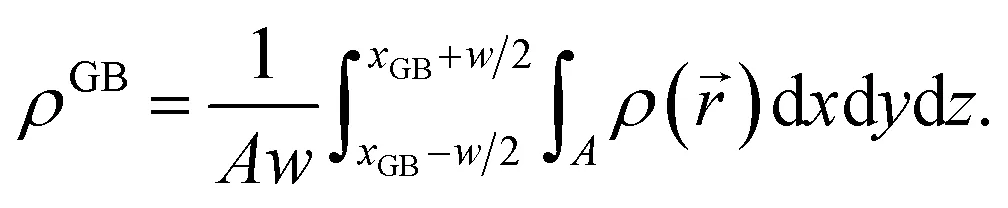
where *w* = 0.2 Å is the bin size for saving the continuous coarse-grained atomic density field. All grain boundary resolved scalar quantities below use the same slab.

Nearest neighbor coordination numbers are obtained from a bulk-referenced cutoff at the first minimum of the bulk radial distribution function, yielding *Z*_1_(*R*_*I*_) at each atom position. We form the grain boundray plane average17
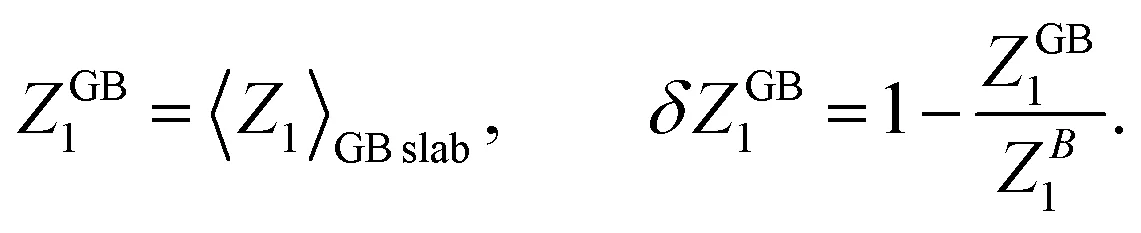
with *Z*_1_^*B*^ = 8. *Z*_2_ is computed similarly, with *Z*_2_^*B*^ = 6.

To quantify spacing effects, we use eqn (12) and (13) with effective shell weights *φ̃*_*k*_. First, we compute the Voronoi volume for each atom, that is, for the BCC structure, the corresponding 2nd shell volume. Using this volume, we compute an equivalent radius *R̃*_2_ and evaluate the same Gaussian kernel *φ̃*_2_. Using the above-computed data and eqn (12), we compute 
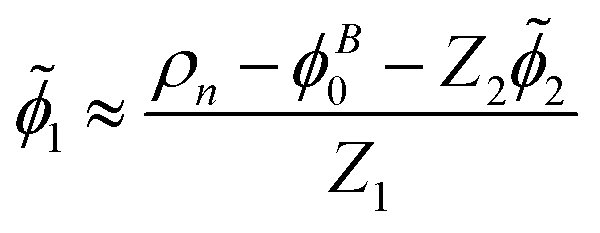
 for each atom. As *R̃*_*k*_ is obtained directly from the spatial coordinates *r⃑* of neighboring atoms—rather than from a topological count of given atoms in that shell—the shell volume is defined by midpoints between successive neighbor distances. In this way, the pair (*Z*_*k*_, *φ̃*_*k*_) provides a natural separation between topology (coordination counts) and geometry (interatomic spacing).

### 5.3 Computing configurational entropy from atomic density and from shell descriptors

We compute configurational Shannon entropies from spatial per-atom distributions obtained from our analyses. Note that these quantities should not be interpreted as full thermodynamic entropies. Rather, they quantify the disorder associated with the spatial distribution of a given descriptor in each system, here including the grain boundary, without accounting for temporal, vibrational, or finite-temperature contributions. Nevertheless, they provide a useful measure for the present comparative analysis of grain boundary descriptors. We thus can compute this quantity per unit area of the grain boundary. For every property *X*,18
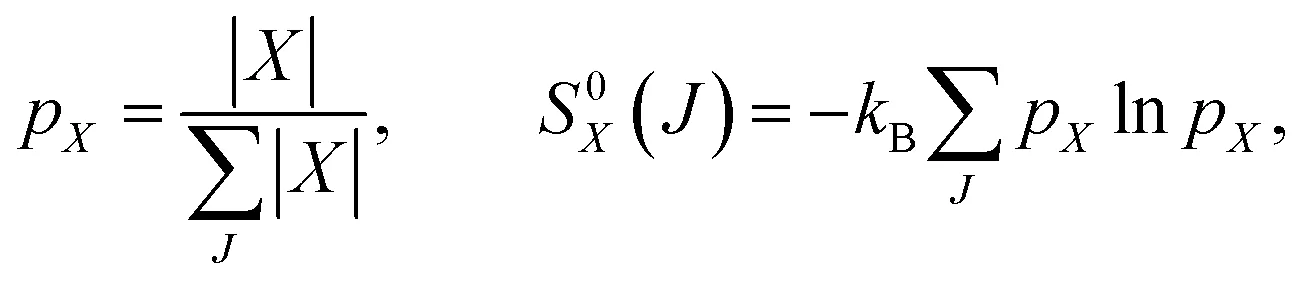
where *X ε* (*ρ*_*n*_, *ρ*_*n*_^*B*^, *Z*_1_, *Z*_1_^*B*^, *φ̃*_1_, *φ̃*_1_^*B*^, *Z*_2_, *Z*_2_^*B*^, *φ̃*_2_, *φ̃*_2_^*B*^), and *J* indicates the atoms in the whole system (*J* = *I*) or a bulk subsystem (*J* = *I*^*B*^). Precisely, *I* denotes ALL atoms (excluding certain boundary atoms in the non-periodic part of the simulation box) and *I*^*B*^ indicates bulk atoms away from the grain boundary plane (±10 Å) and also non-periodic boundaries along the *x* axis. Thus, we compute the grain boundary excess entropy19
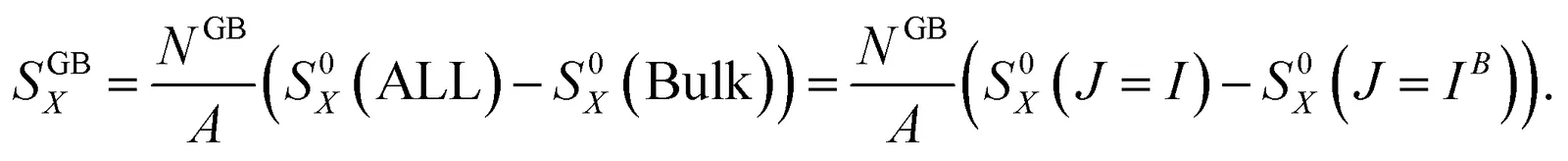
For example
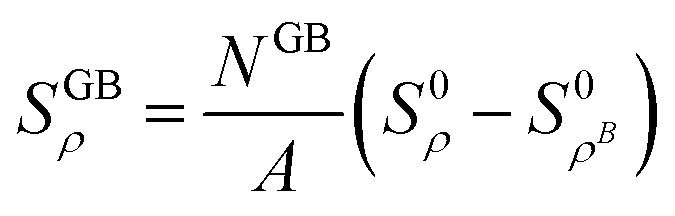
, where *N*^GB^ and *A* are the number of atoms and the area of the grain boundary.

The contributions due to individual descriptors (
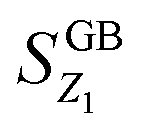
, 
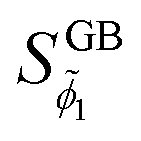
, 
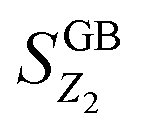
, 
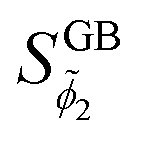
) are shown in Fig. S3 and S4 in the SI, revealing that the entropy contributions due to coordination deficiency 
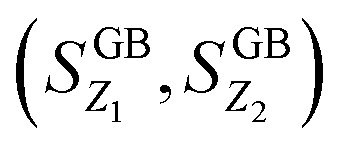
 are much smaller than the entropy due to disturbance in the atomic spacing 
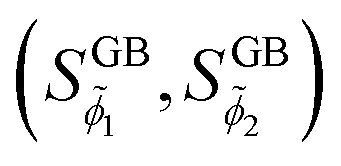
.

### 5.4 Computing the sum entropy and correlation analysis

Entropies can be summed in specific cases where they represent independent contributions to the total entropy of a system, such as (i) when considering the entropy of multiple subsystems that do not interact with each other, (ii) when the entropies come from different physical sources (*e.g.*, vibrational, magnetic, *etc.*), or (iii) as in statistical mechanics, when the system is partitioned into independent entities (*e.g.*, particles' position and momentum, their energy states, *etc.*). In those specific cases, the total entropy of the system is the sum of the entropies of the subsystems, sources, or entities.

For our eqn (12), despite the apparent randomness of the grain boundary environment, it is conceivable that *Z*_1_, *Z*_2_, *φ̃*_1_, and *φ̃*_2_ are correlated variables and not strictly independent, constrained by the fundamental coordination-distance compatibility, subject to free-energy minimization. As a result, the corresponding entropy contributions are expected to exhibit partial correlations. The relevant question is therefore not whether the descriptors are correlated, but to what extent. Consequently, the sum entropy cannot be treated as strictly additive, *i.e.*,20
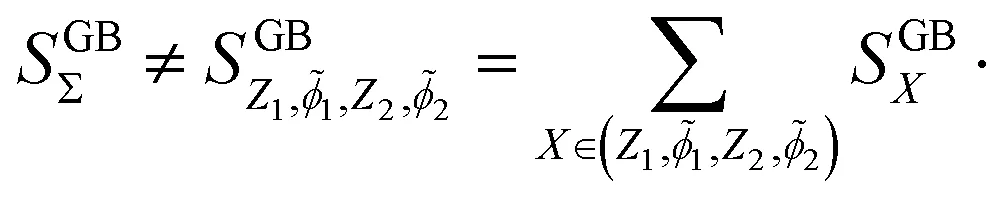


In order to correct for the “overlapping information” among these parameters, we must account for the coupling effects such that21
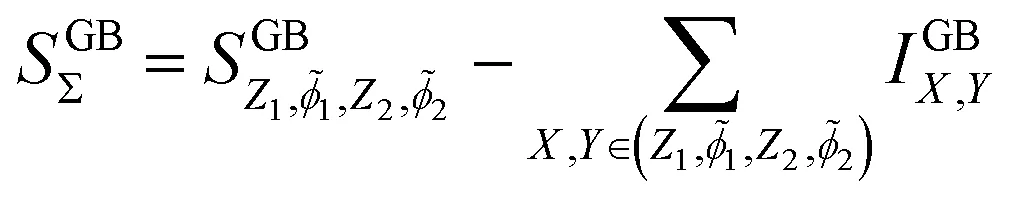
where *I*^GB^_*X,Y*_ represents the mutual entropy among (six) pairs of parameters. This mutual entropy can be defined as

22 *I*^GB^_*X,Y*_ = *S*^GB^_*X*_ + *S*^GB^_*Y*_ − *S*^GB^_*X,Y*_,

with *S*^GB^_*X,Y*_ being the joint entropy and23
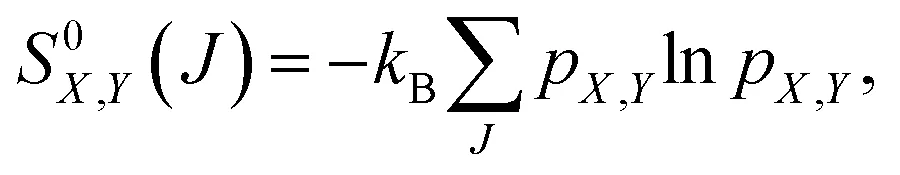
for which eqn (19), to compute the grain boundary contribution, applies.

It is worth remarking that careful inspection of eqn (21) tells us that in subtracting pair-coupling, triple- or quadruple-coupling of the parameters may be double- or triple-counted. These double or triple countings must be computed and added back to the entropy, in order to correctly define the unique parameter space and the corresponding sum entropy:24
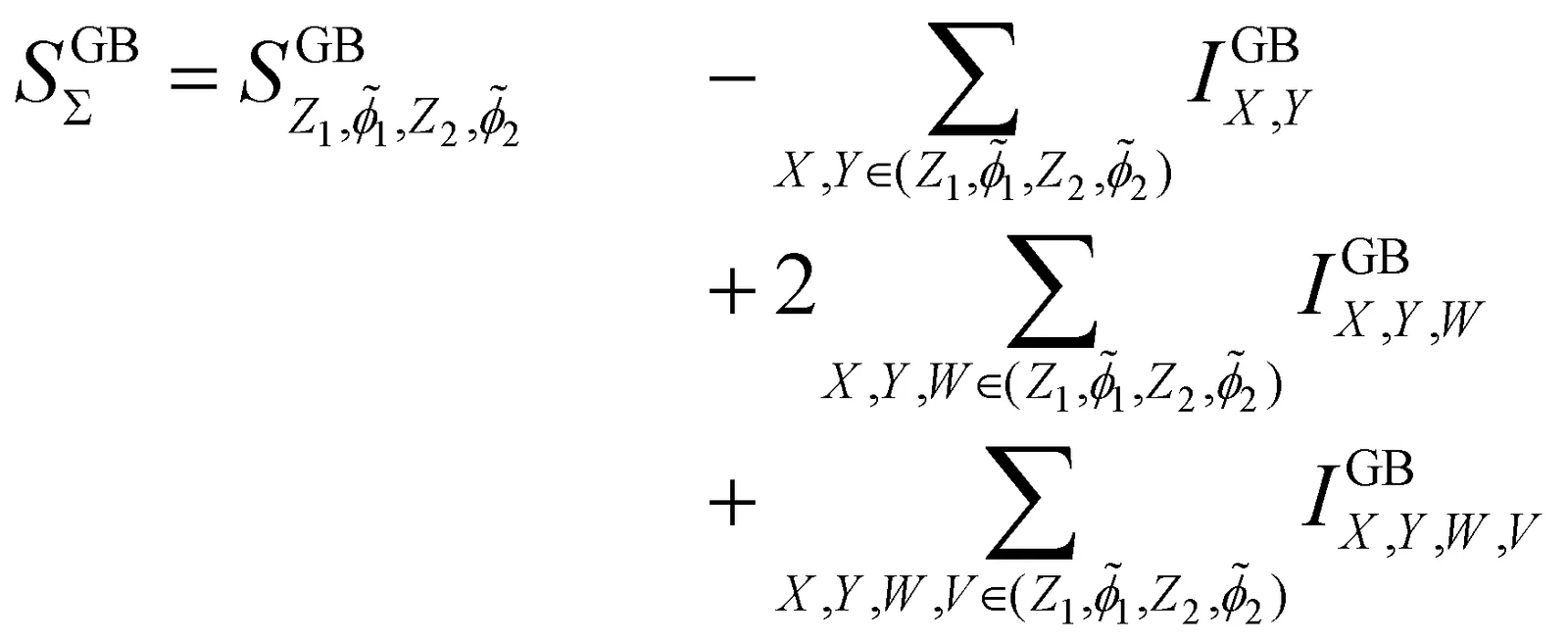
in which *I*_*X*,*Y*,*W*_ and *I*_*X*,*Y*,*W*,*V*_ are triple and quadruple entropies and must be defined analogously to eqn (22). In the following, for the sake of simplicity, we stay with eqn (21) and the assumption of having only non-overlapping pair correlations.

The joint entropy can be computed if we know the joint probability distribution, *p*_*X*,*Y*_, of the configurations of *X* and *Y*, *i.e.*, how they vary together in the system:

25 *p*_*X,Y*_ = *p*(*X* and *Y*)

Knowing only the marginal distributions, however, does not uniquely determine the joint distribution: many different joint distributions can share the same marginals, even though they are bounded by the Fréchet–Hoeffding limits. On top of the marginal distributions, we need the so-called dependence structure, the second derivative of which (Sklar's theorem) formally enables defining the joint probability.

If we know about the full family of conditionals, we can compute the conditional probability *p*_*Y*|*X*_ and, subsequently, the joint probability

26 *p*_*XY*_ = *p*_Y|X_·*p*_*X*_

For our grain boundaries, however, we neither know the dependence structure nor the full family of conditionals, which requires guaranteeing that *∑*_*j*_*p*_*Y*|*X*_(*j*|*i*) = 1 for each *X* = *i*, and *vice versa*. Our analyses (not further discussed here) show that the dependence structure cannot be approximated based on our current atomistic simulations, but requires extensive, systematic studies to capture the full family of conditionals.

An idea pursued was that the interdependence of atomic coordinations and spacings can be evidenced by studying the cross-correlation among them. To rationalize the empirical relation 
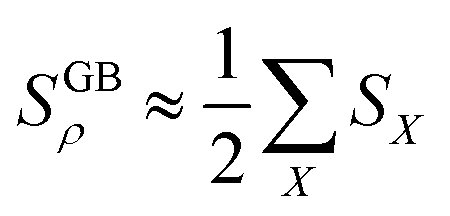
, with *X* = (*Z*_1_, *φ̃*_1_, *Z*_2_, *φ̃*_2_), we analyze the Pearson cross-correlations among six descriptor pairs *r*(*Z*_1_, *Z*_2_), *r*(*Z*_1_, *φ̃*_1_), *r*(*Z*_1_, *φ̃*_2_), *r*(*Z*_2_, *φ̃*_1_), *r*(*Z*_2_, *φ̃*_2_), *r*(*φ̃*_1_, *φ̃*_2_) for each grain boundary,27
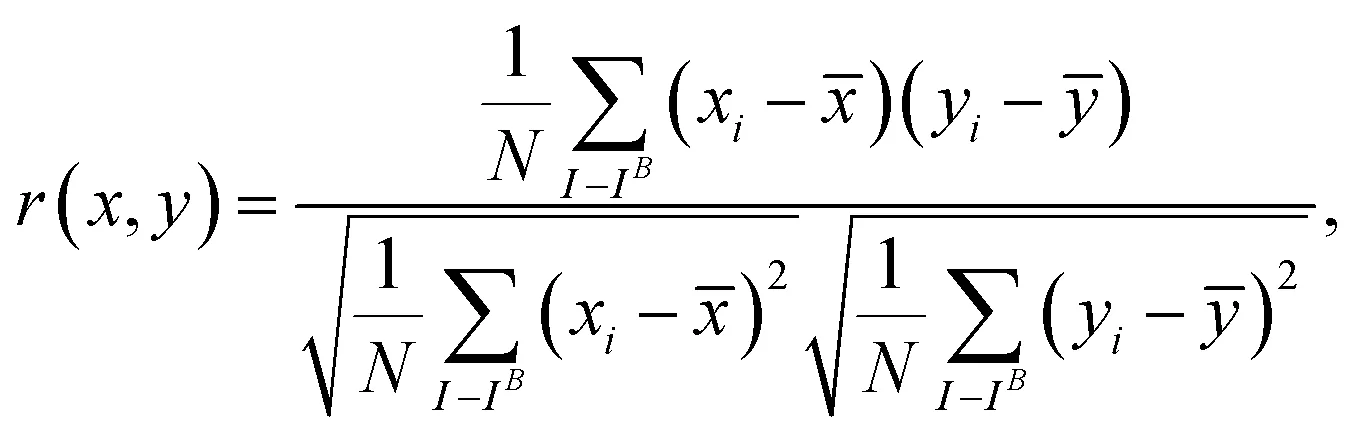
with *N* the number of atoms in the corresponding *I* − *I*^*B*^ region.

Fig. S7(a–f) in the SI Material depicts the cross-correlation computed for all grain boundaries, showing that.

• *Z*_1_ − *φ̃*_1_: tightly negative (cluster near −0.9). First shell behaves like one DOF: more 1st neighbors (up to its ideal number 8) means shorter mean 1st-shell distance.

• *Z*_2_ − *φ̃*_2_: strongly negative (often −0.7 to −0.9). Same story for the second shell.

• *Z*_1_ − *φ̃*_2_: strikingly positive (often +0.7 to +0.95). Pack the 1st shell and the 2nd shell shifts outward (volume/elastic response).

• *Z*_2_ − *φ̃*_1_: positive but weaker. Cross-shell spillover but not as tight.

• *φ̃*_1_ − *φ̃*_2_: mostly negative. When the inner shell stretches, the outer tends to contract, and *vice versa*.

• *Z*_1_ − *Z*_2_: mostly negative (often −0.2 to −0.5). If the 1st shell is over-coordinated, the 2nd tends to be under-coordinated.

This pattern indicates that, within each shell, the descriptor pairs (*Z*_1_, *φ̃*_1_) and (*Z*_2_, *φ̃*_2_) are highly redundant and therefore represent nearly single effective modes. Across shells, however, the correlations reveal coupling without full collapse. This means that the four-dimensional descriptor variability is approximately rank-2, with two dominant principal components and two strongly suppressed (correlated) residual components. Thus, within each shell *k* ∈ {1, 2} we can write28
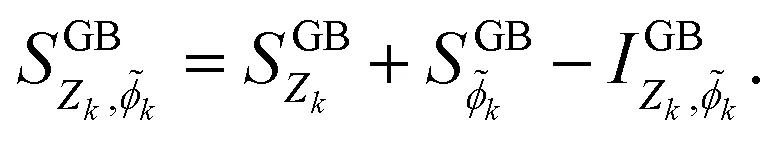
The plots suggest that (*Z*_*k*_, *φ̃*_*k*_) is almost one direction (large |*r*|), so the “overlap” term is large. Expressing this overlap as a fraction *λ*_*k*_ of the naive sum,29
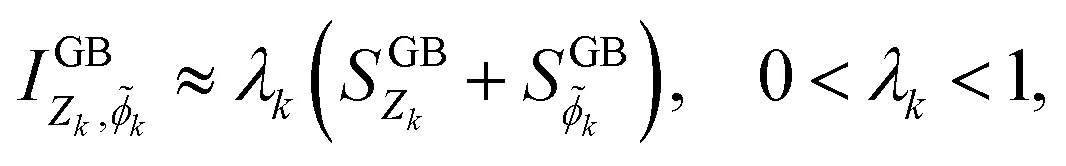
then eqn (28) becomes30
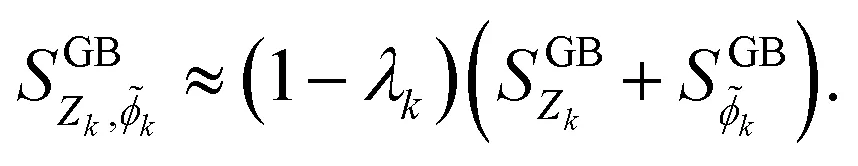


Empirically we see 
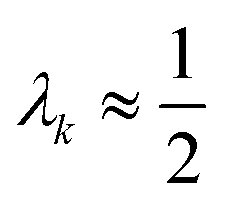
 for both shells, which is exactly the statement that the joint per shell is roughly half the naive sum:31
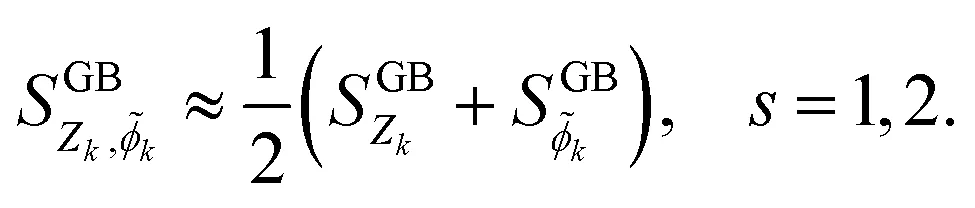


Now, for the four-tuple, keeping only the leading shell blocks and (at first pass) dropping the residual coupling between the two shells, this means32
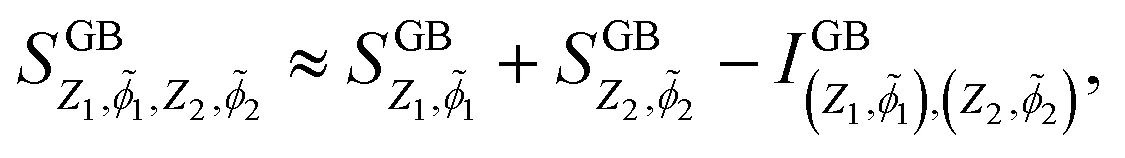
and if the cross-shell coupling term on the right is small, one can essentially add the two shell joints:33
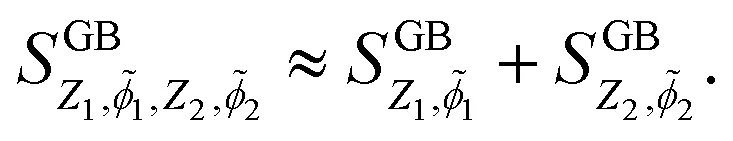
Substituting eqn (31) into eqn (33) gives the “half-sum” at the level of grain boundary entropies:34
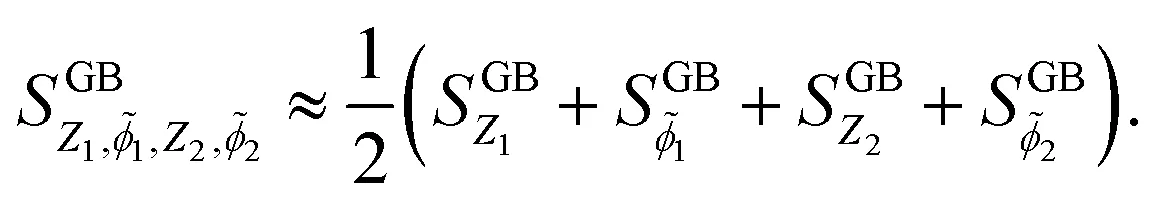
Our results indicate that 
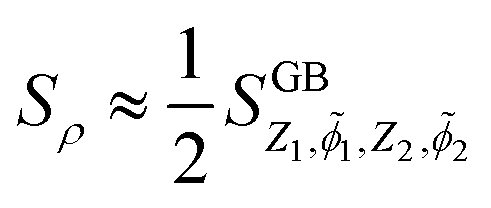
 captures the entropy associated with these two dominant modes, while the factor 1/2 reflects the effective redundancy among the descriptors arising from their correlation structure. Extensive details on all entropy-related calculations will be added soon as SI.

Sanity check for eqn (31)Although it is difficult to prove, one can assume a zero-mean bivariate normal with unit variances and correlation *r*:35
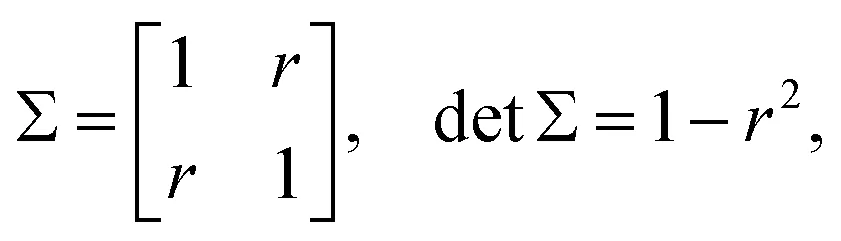
which simplifies the differential entropy of a *k*-variate normal to36
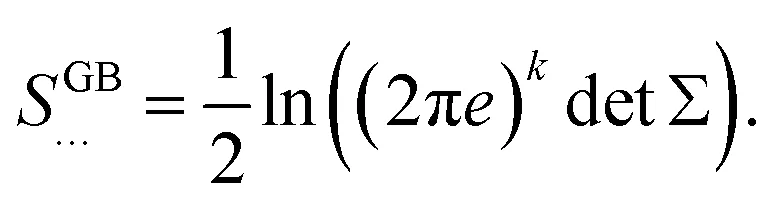
with *k* = 2 and using eqn (35),37
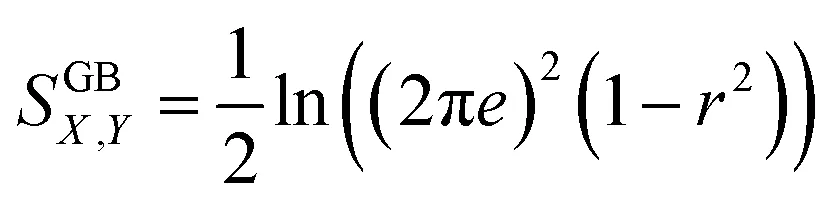
38
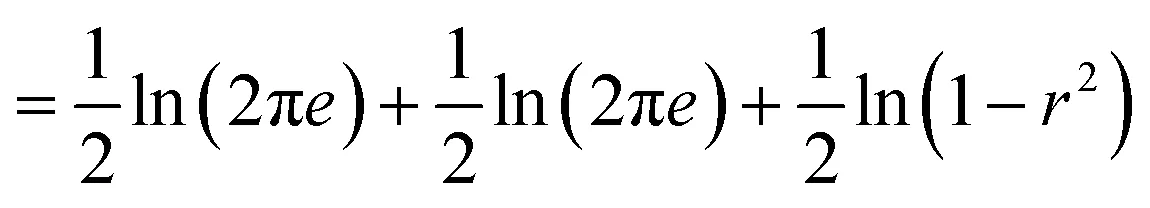
39
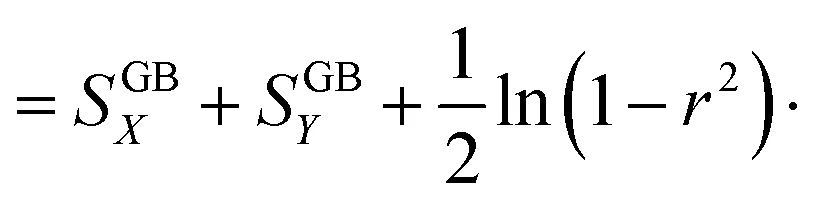
which is due to 
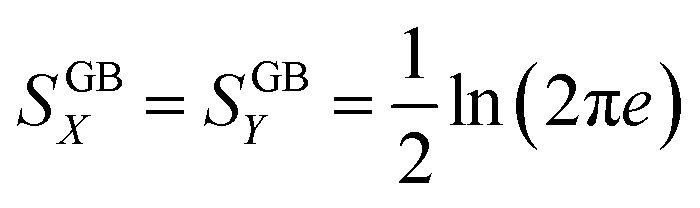
. Equivalently, by the chain rule and Gaussian conditioning,

40 *S*_*X*,*Y*_^GB^ = *S*_*X*_^GB^ + *S*_*Y*|*X*_^GB^,

41
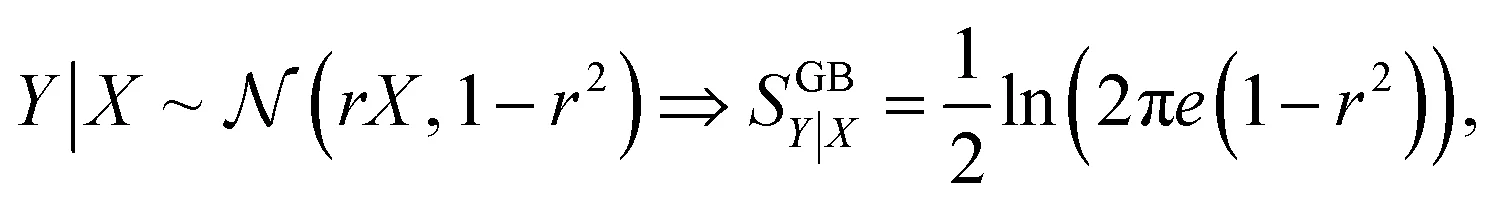
42
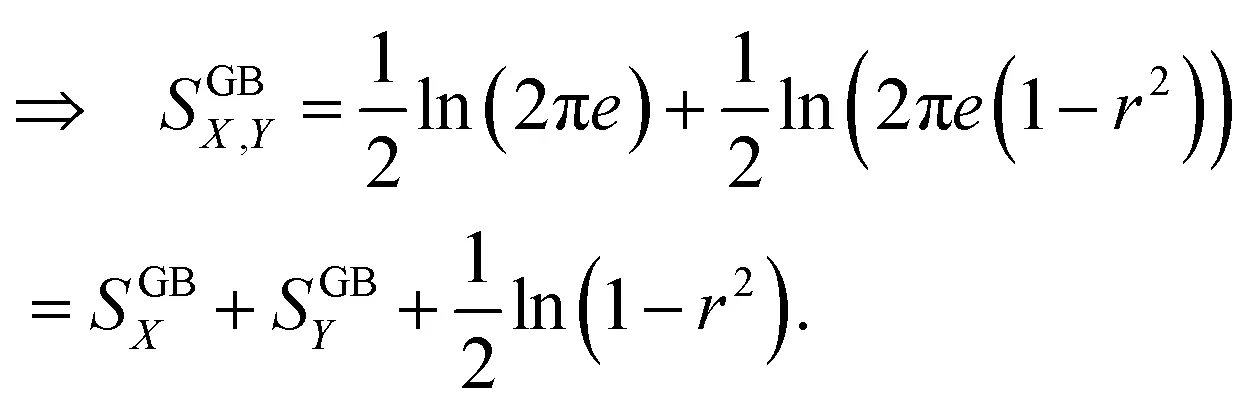


Now, asking for 
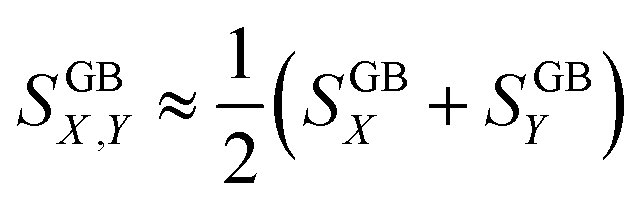
 leads to

43 ln(1 − *r*^2^) ≈ −(*S*_*X*_^GB^ + *S*_*Y*_^GB^),

which gives44
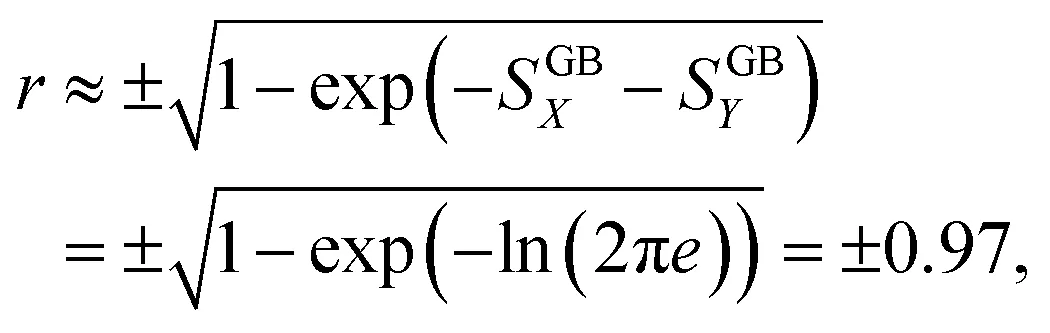
*i.e.*, |*r*| is very close to 1 (at least with standardized marginals), which is consistent with what we indeed measure for (*Z*_1_, *φ̃*_1_) and (*Z*_2_, *φ̃*_2_) correlations.

As a final remark, one should note that the correlation among *Z*_1_, *Z*_2_, *φ̃*_1_, and *φ̃*_2_ is not, strictly speaking, a grain boundary property, but depends on the specific sub-boundary's defect structure. In the simplest scenario, where a grain boundary is composed of a single structural motif, one may assume that the coefficient 1/2 represents the exact limiting value. Exploring the quality of this coefficient remains the subject of revealing and likely delightful future research.

## Conflicts of interest

The authors declare no competing financial interests.

## Supplementary Material

RA-OLF-D6RA05403J-s001

## Data Availability

Further data and codes of this study are available from the corresponding author. Supplementary information (SI) is available. See DOI: https://doi.org/10.1039/d6ra05403j.
