# Supplementary material for: On the relationship and distinction between atomic density and coordination number in describing crystal defects
Source: RSC Adv. 2026 Oct 23;16(48):65516–27. doi: 10.1039/d6ra05403j (PMC13692009; doi:10.1039/d6ra05403j)
Supplement: Supplementary file 1 [file RA-016-D6RA05403J-s001.pdf]

Supplementary Information (SI) for RSC Advances.
This journal is © The Royal Society of Chemistry

Supplementary Material for

On the Relationship and Distinction Between Atomic Density and Coordination Number in Describing Crystal Defects

Reza Darvishi Kamachali*[,1,2] and Theophilus Wallis[1]

[1]Federal Institute for Materials Research and Testing (BAM), 12205 Berlin, Germany
[2]Institute of Materials Physics, University of Münster, 48149 Münster, Germany

In this section, we present additional results that examine the relationship and distinctions between atomic density and coordination number in grain boundary characterization. Each figure is accompanied by a comprehensive caption, while further detailed discussion is provided in the main paper.

The following are presented:

- **Figure S1**: Representative grain boundary examples with corresponding descriptor distributions at the interface.
- **Figure S2**: Representative grain boundaries illustrating the coordination number variations.
- **Figure S3**: Grain boundary excess entropy per unit area descriptors plotted as a function of the coordination deficit ($8–Z_1^{GB}$) at the GB plane.
- **Figure S4**: Grain boundary excess entropy per unit area descriptors plotted as a function of the density deficit ($1 - \rho^{GB}$) at the GB plane.
- **Figure S5**: Grain boundary energy versus sum configurational entropy per unit area $\frac{1}{2}\, S^{GB}_{Z_1,\tilde{\phi}_1,Z_2,\tilde{\phi}_2}$ and areal configurational entropy per unit area from the density field S^{GB}_{ρ}.
- **Figure S6**: First neighbor coordination entropy versus sum entropy.
- **Figure S7**: Pearson cross-correlations among six descriptor pairs.
- **Figure S8**: Legend depicting grain boundary classification groups.

*Corresponding author: reza.kamachali@bam.de; reza.kamachali@gmail.com

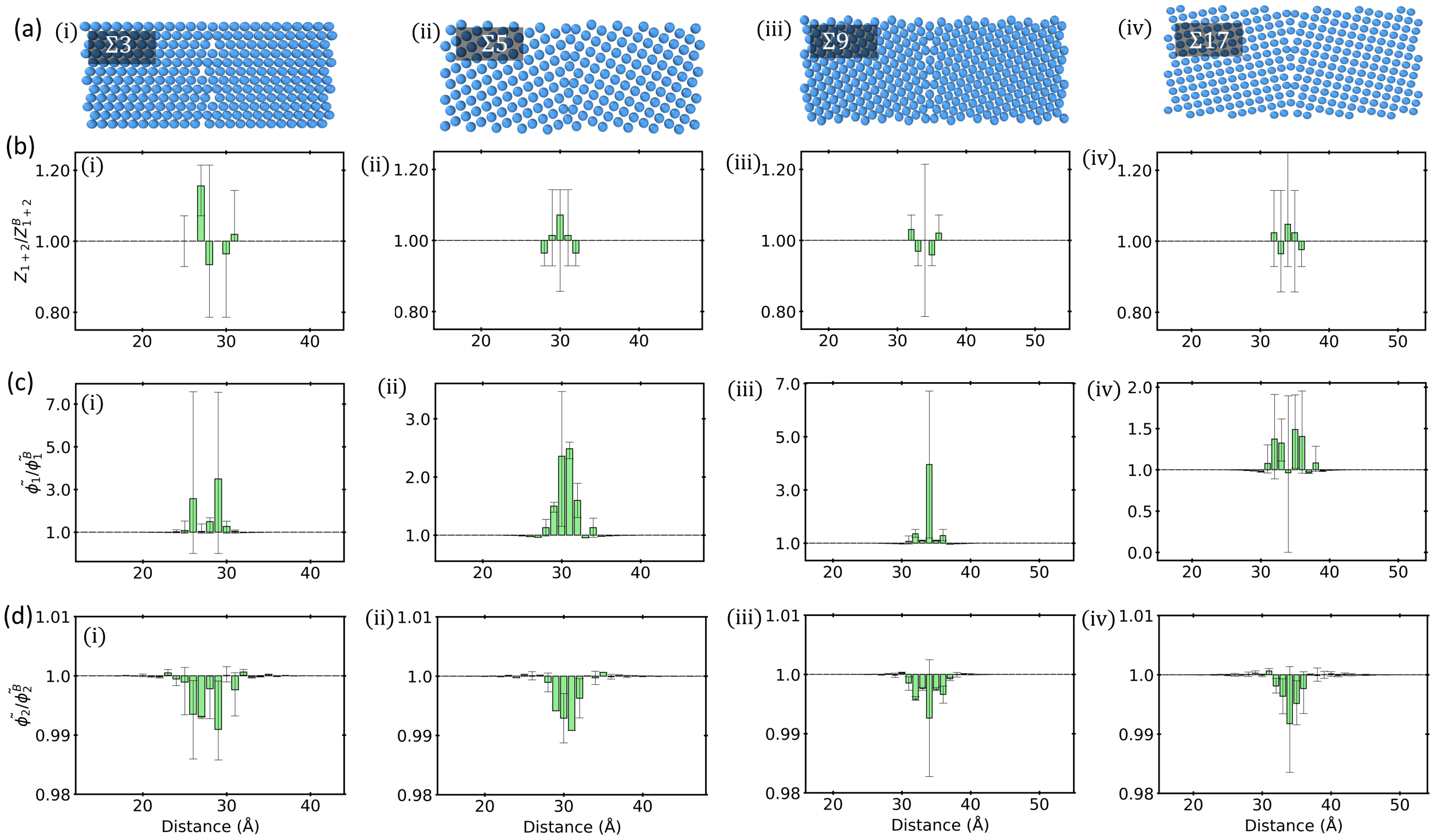

Figure S1: Illustrative examples of grain boundaries. (a) atomistic structures for example Σ3, Σ5, Σ9 and Σ17 boundaries. The profiles of (b) $Z_{1+2}(x)/Z^B_{1+2}$, (c) $\tilde{\phi}_1(\mathrm{x})/\phi^B_1$, (d) $\tilde{\phi}_2(\mathrm{x})$ /ϕ^B_2 across the GB are shown, revealing the descriptor distribution at the interface. Here the descriptor quantities within the bulk Z^B_2, ϕ^B_1 and ϕ^B_2 are given as 14, 0.00713 and 0.00206 respectively. Here, $Z_{1+2} = Z_1 + Z_2$ denotes the cumulative coordination number through the first and second coordination shells. For bulk BCC Fe, $Z^B_1 = 8$ and $Z^B_2 = 6$, giving $Z^B_{1+2} = 14$. The bulk values of ϕ^B_1 and ϕ^B_2 are 0.00713 and 0.00206, respectively. In panels (b) to (d), the error bars denote the variation of the respective quantity within the corresponding yz-slice, sweeping across the GB plane along the x-axis.

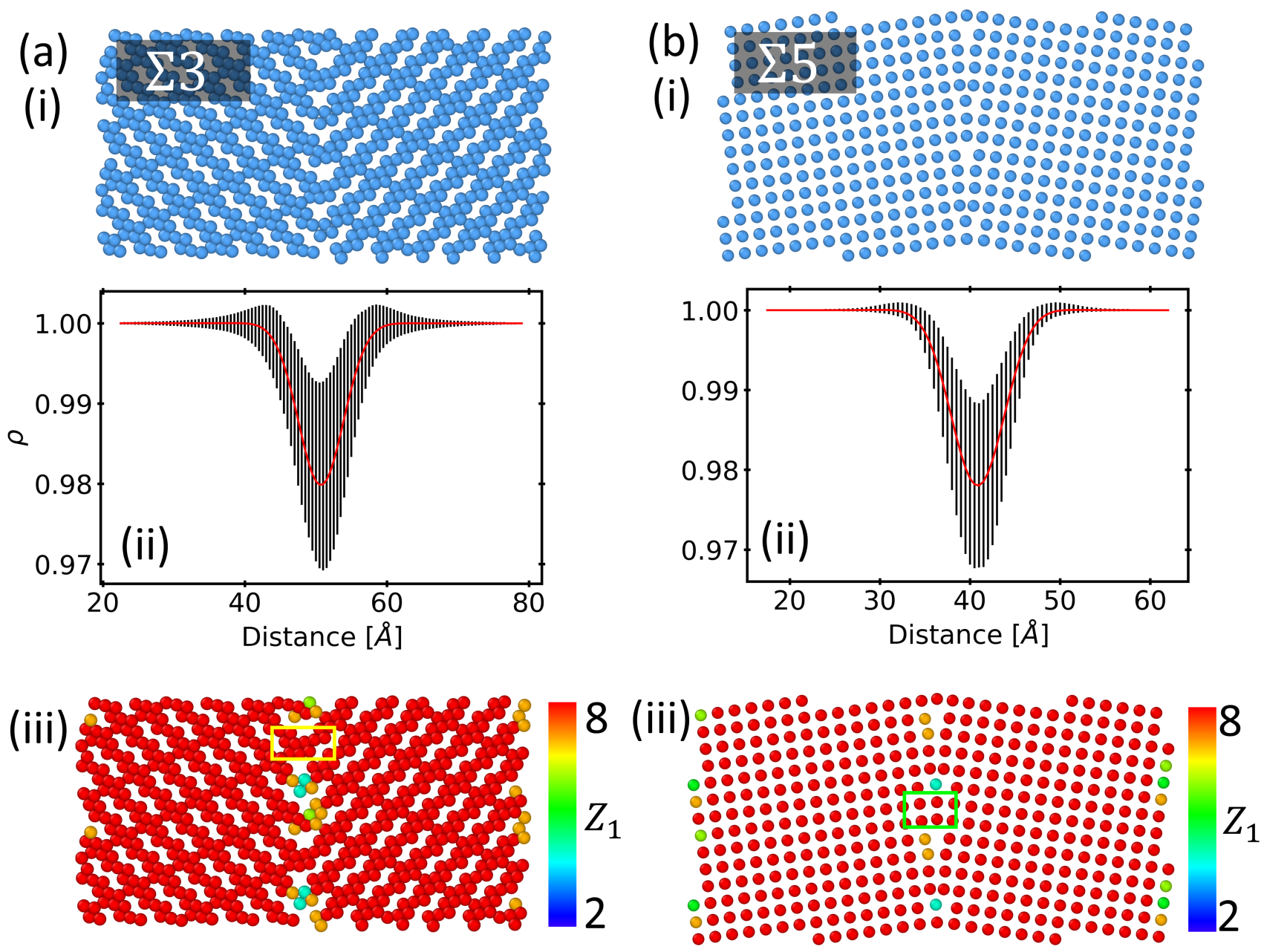

Figure S2: Representative grain boundaries illustrating local coordination number variations. (a) Σ3 and (b) [100] tilt (Σ5) GB. For each boundary, (i) shows the relaxed atomic structure, (ii) the coarse-grained atomic density profile, and (iii) the atomic structure colored according to the first neighbor coordination number. In both cases, the density field exhibits a spatially continuous depletion at the GB, whereas the coordination number variations depend on the specific atomic arrangement. In particular, atoms within the density depleted region can retain the bulk BCC coordination, $Z_1 = 8$, demonstrating that changes in interatomic spacing can occur without necessarily altering first neighbor connectivity. The highlighted boxes indicate representative interfacial regions illustrating this behavior.

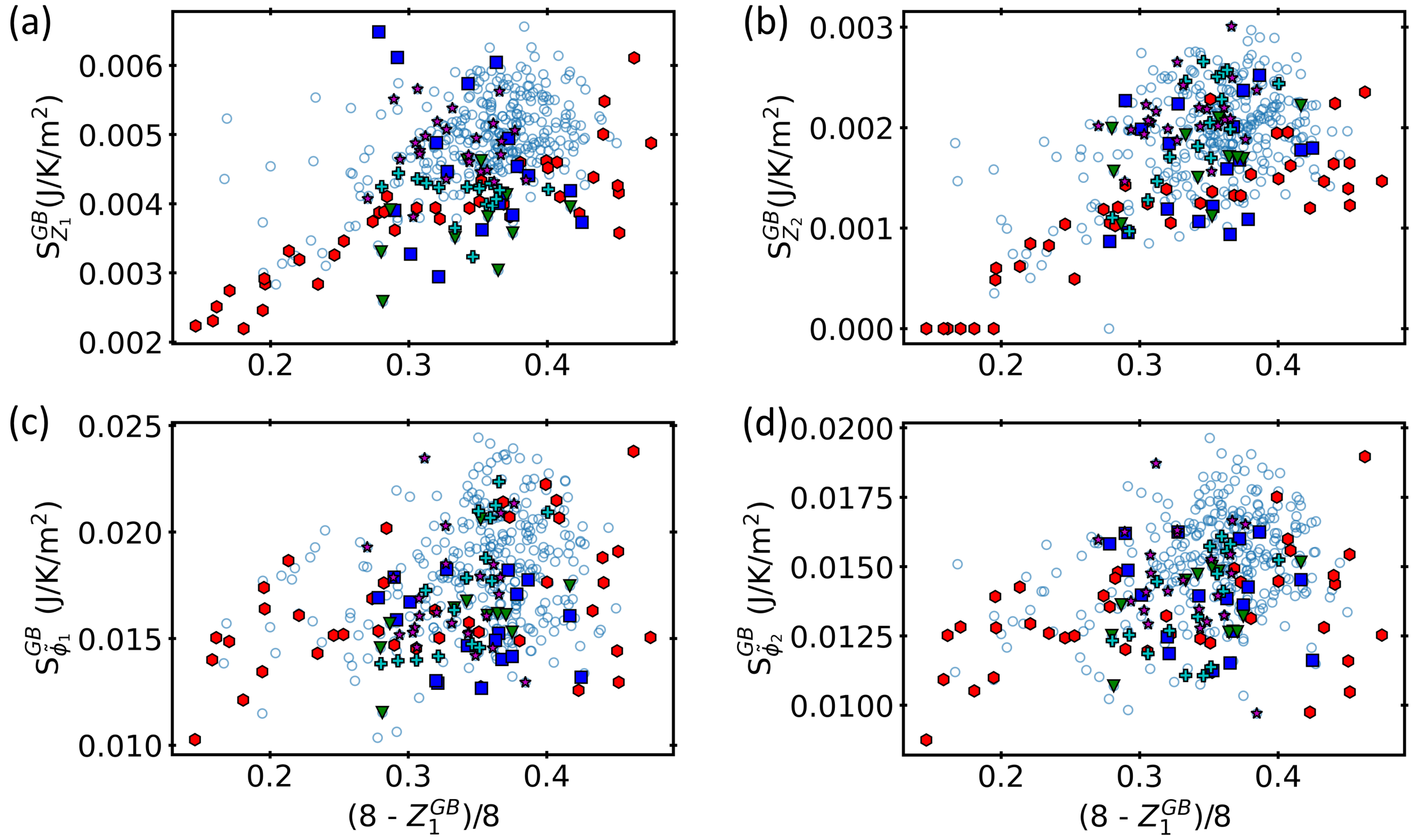

Figure S3: Grain boundary excess entropy per unit area. The grain boundary excess entropy is evaluated in terms of (a) the number of first-nearest neighbors, (b) the number of second-nearest neighbors, (c) the normalized Gaussian contribution from the first shell, and (d) the normalized Gaussian contribution from the second shell. All quantities are plotted as a function of the coordination deficit ($8–Z_1^{GB}$) at the GB plane. Grain boundaries are grouped by parent plane family, as shown in the legend in Fig. S8.

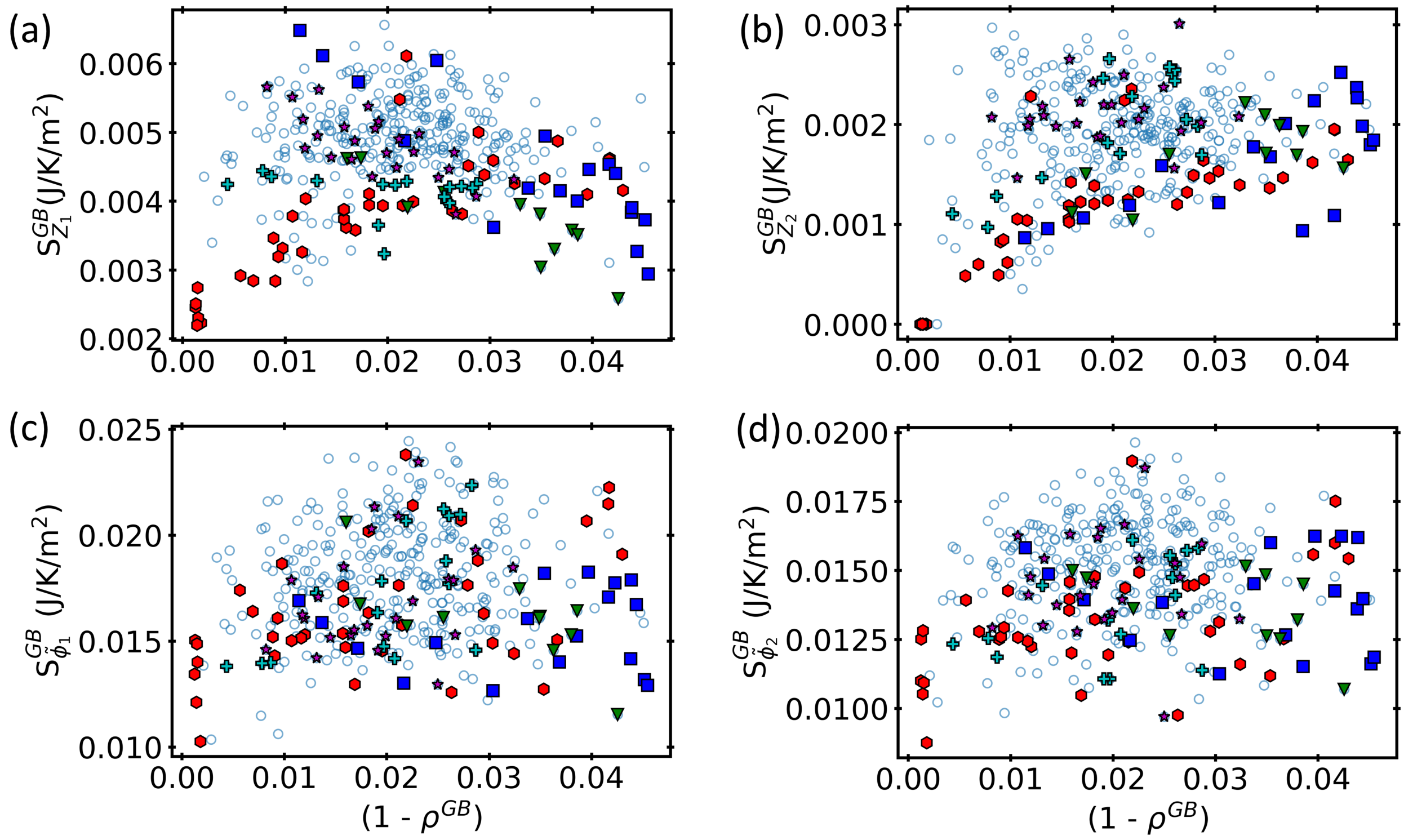

Figure S4: Grain boundary excess entropy per unit area. The grain boundary excess entropy is evaluated in terms of (a) the number of first-nearest neighbors, (b) the number of second-nearest neighbors, (c) the normalized Gaussian contribution from the first shell, and (d) the normalized Gaussian contribution from the second shell. All quantities are plotted as a function of the density deficit (1 - ρ^{GB}) at the GB plane. Grain boundaries are grouped by parent plane family, as shown in the legend in Fig. S8.

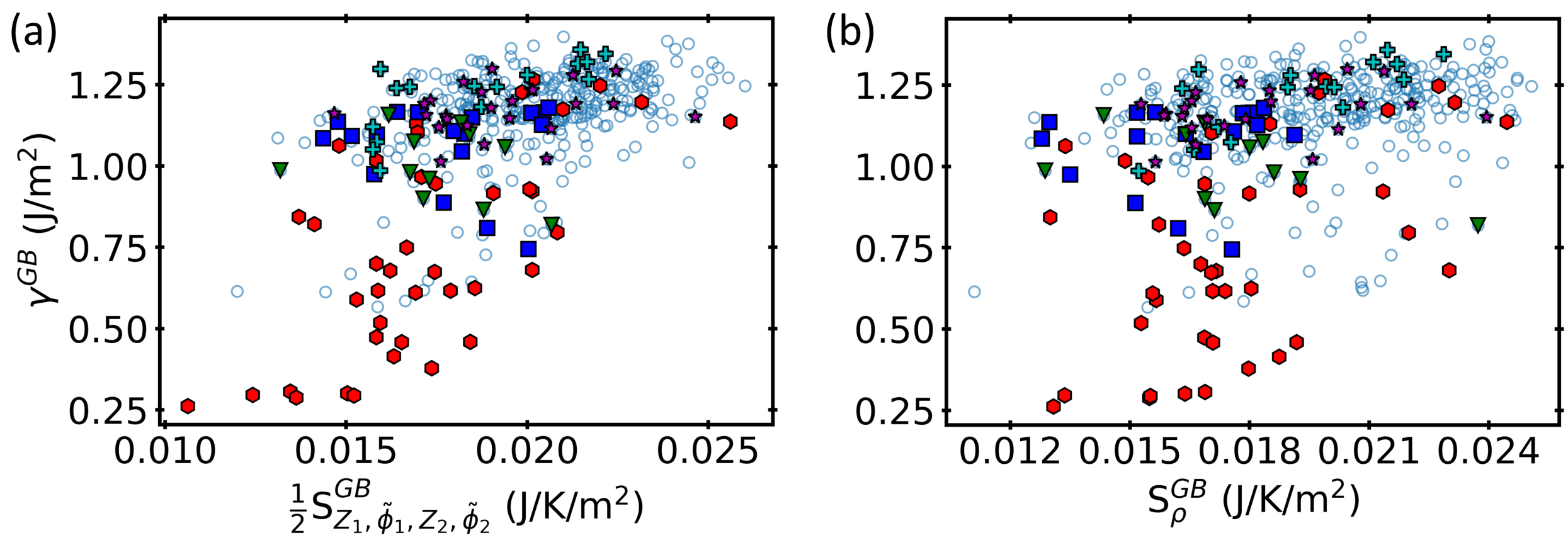

Figure S5: Relationship between grain boundary energy and configurational entropy. (a) The GB energy γ^{GB} increases with increasing sum configurational entropy per unit area $\frac{1}{2}S^{GB}_{Z_1,\tilde{\phi}_1,Z_2,\tilde{\phi}_2}$; (b) the same GB property versus areal configurational entropy from the density field S^{GB}_{ρ} reveals a similar relationship. Grain boundaries are grouped by parent plane family, as shown in the legend in Fig. S8.

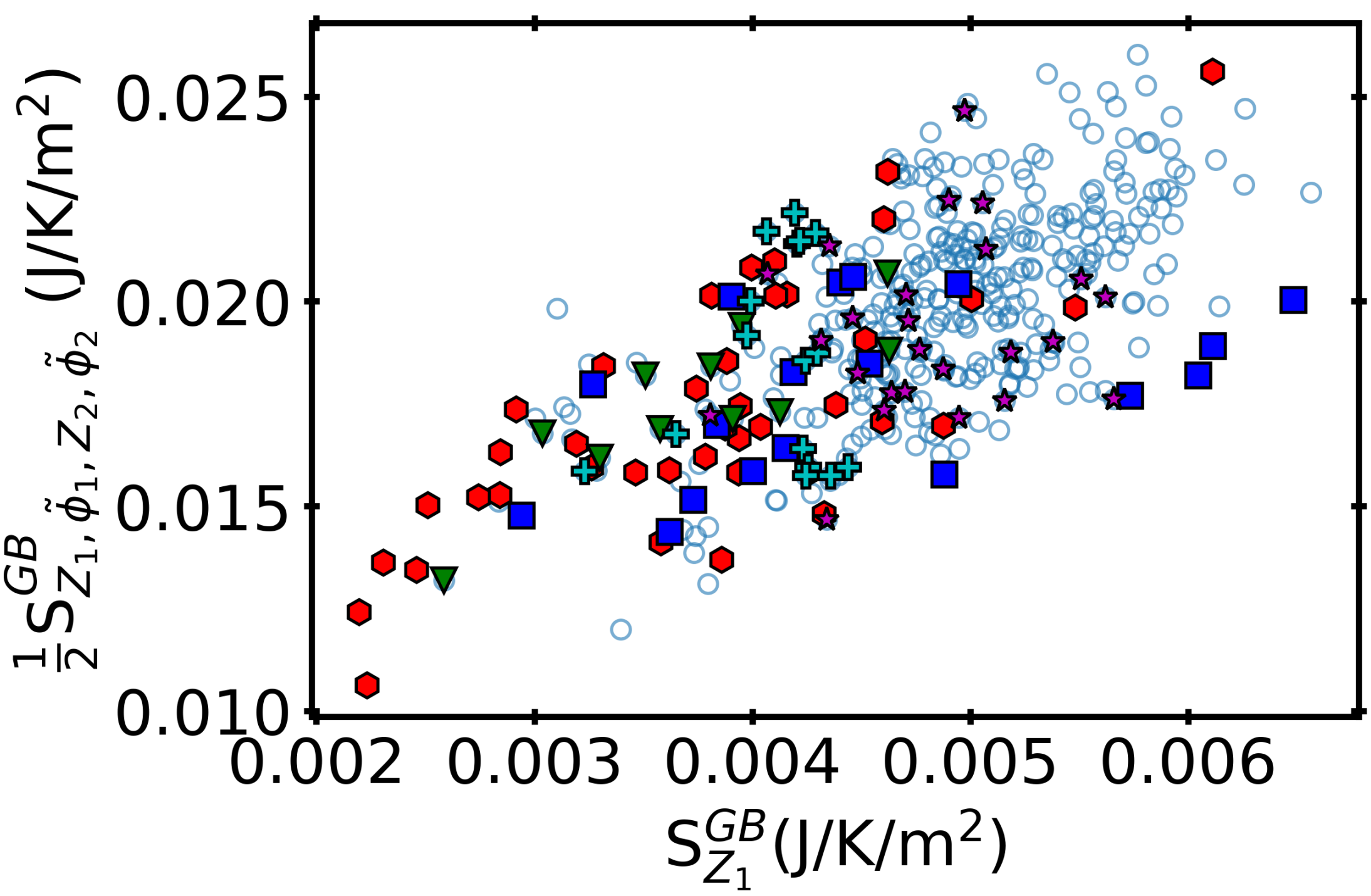

Figure S6: First neighbor coordination entropy versus sum entropy. Comparison of the first neighbor coordination entropy $S^{GB}_{Z_1}$ with the sum entropy $\frac{1}{2}S^{GB}_{Z_1,\tilde{\phi}_1,Z_2,\tilde{\phi}_2}$ across the investigated grain boundaries. The two entropy measures exhibit an overall loose relationship with appreciable scatter. Grain boundaries are grouped by parent plane family, as shown in the legend in Fig. S8.

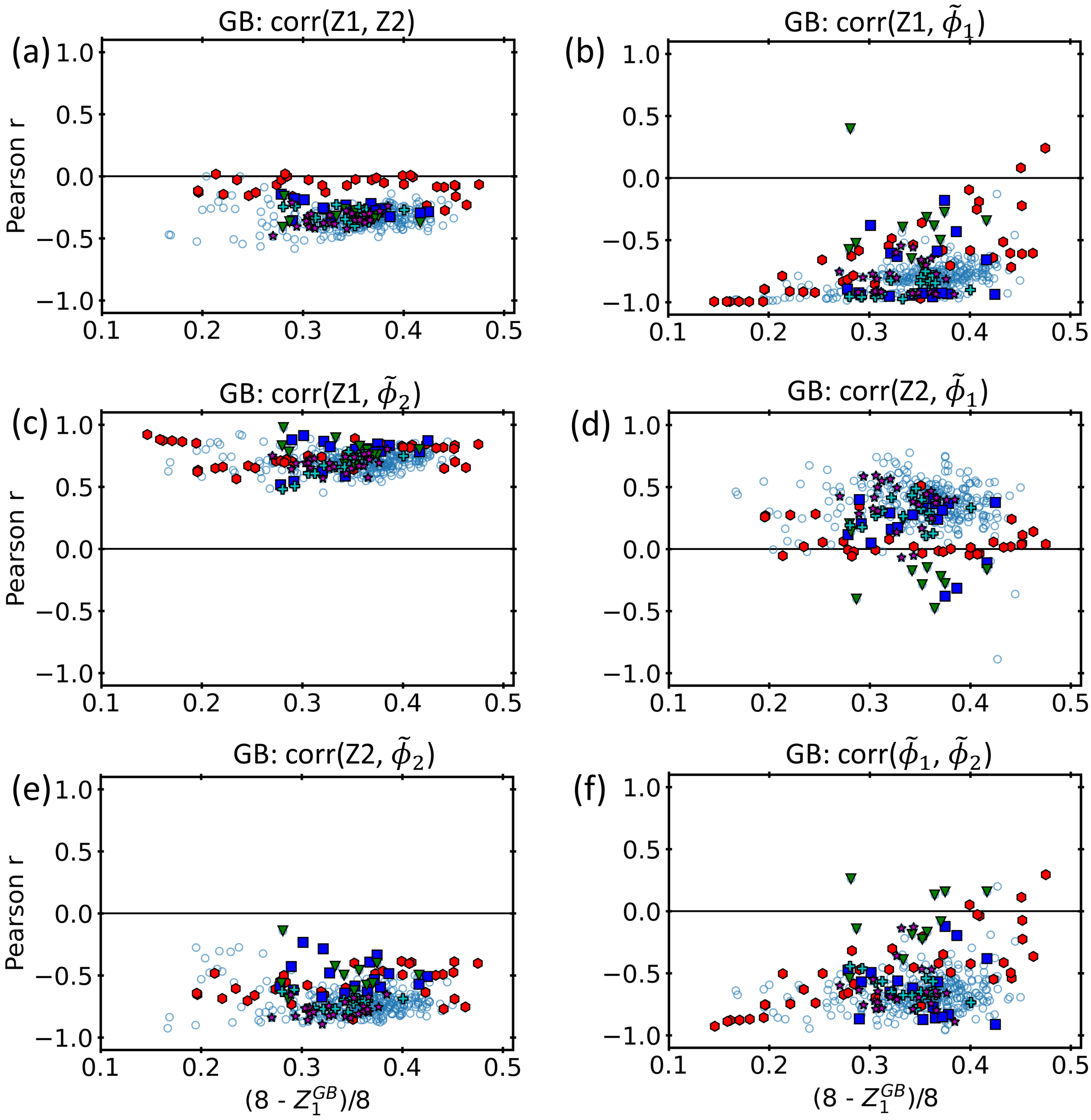

Figure S7: Pearson cross-correlations among six descriptor pairs. Pearson cross-correlation among (a) r(Z_1, Z_2), (b) r(Z_1, $\tilde{\phi}_1$), r(Z_1, $\tilde{\phi}_2$), (c) r(Z_2, $\tilde{\phi}_1$), (d) r(Z_2, $\tilde{\phi}_1$, (e) r(Z_2, $\tilde{\phi}_2$, and (f) r($\tilde{\phi}_1$, $\tilde{\phi}_2$). All descriptor pairs are plotted as a function of the coordination deficit $(8 - Z_1^{GB})/8$ at the GB plane. A strong correlation dominates the descriptor pairs r(Z_1, $\tilde{\phi}_1$) and r(Z_2, $\tilde{\phi}_2$). Grain boundaries are grouped by parent plane family, as shown in the legend in Fig. S8

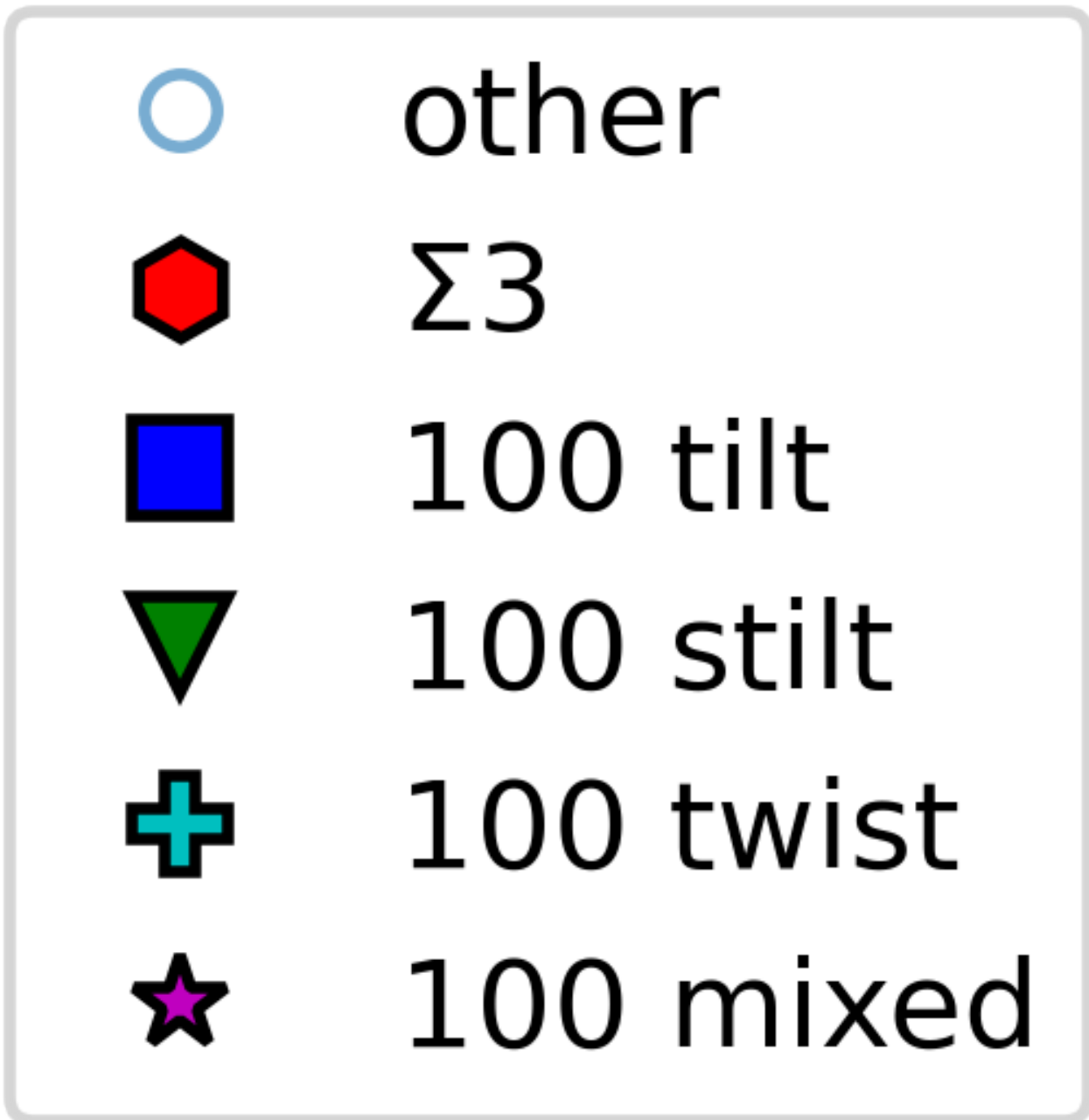

Figure S8: Legend. Legend depicting grain boundary classification groups
